# Deterring delinquents with information. Evidence from a randomized poster campaign in Bogotá

**DOI:** 10.1371/journal.pone.0200593

**Published:** 2018-07-19

**Authors:** Enzo Nussio, Ervyn Norza Céspedes

**Affiliations:** 1 Center for Security Studies, ETH Zurich, Switzerland; 2 Departamento de Ciencia Política, Universidad de los Andes, Bogotá, Colombia; 3 Facultad de Psicología, Universidad del Bosque, Bogotá, Colombia; 4 Observatorio del Delito, Policía Nacional de Colombia, Bogotá, Colombia; Bates College, UNITED STATES

## Abstract

In this article, we test whether an isolated information campaign can deter criminals by appealing to their apprehension risk perception. A randomized trial was conducted around 154 high crime housing blocks in Bogotá. With support of the Colombian Police, half of the blocks were exposed to a three month poster campaign reporting the number of “arrests around this street block” and half to a no-treatment control condition. The main outcome measure (total registered crime) and secondary outcome measures (calls to the emergency line for thefts and attacks, and minor wrongdoings) were provided by the Police. Additionally, trust in police, security perception, and police performance perception were measured among residents and workers in the treatment and control areas (N = 616) using a post-treatment survey. Measures were analyzed with linear regression analysis and two-sample t-tests. Over the course of the treatment period, premeditated crime was reduced, while spontaneous crime remained unchanged. Overall levels of crime were not significantly altered. Also, a moderate crime reduction is detectable during the first month of the treatment period. The posters were highly visible (93% of respondents in the treated areas recalled them) and positively received (67% “liked” them). Perceptions of security and police among locals improved, though not significantly. Inherent among residents of Bogotá is a pervasive feeling of impunity and low trust in authorities, making the city a hard test case for an offender-targeted advertising campaign. Initial reductions of crime and overall reductions of premeditated crime are thus noteworthy. These results align with key principles of apprehension risk updating theory.

## Introduction

A credible deterrence threat for delinquents commonly emanates from the physical presence of the police and the related certainty of punishment [1,2]. More generally speaking, as Zimring and Hawkins [3] put it, “the deterrence threat may perhaps best be viewed as a form of advertising.” Deterrence is effective when potential offenders update their risk calculation for committing a crime, specifically the risk of being apprehended [4,5]. Earlier studies have found that publicity for crime prevention schemes in the UK [6], direct communication with gang members and warnings about the legal consequences of their actions in Boston [7], or watching eyes and accompanying warning messages for potential bike thieves [8] are effective forms of deterrence. However, advertising is still “rarely employed as part of routine enforcement activities” [9].

In collaboration with the Colombian Police, we designed a study to test the potential of an offender-targeted campaign, by placing actual apprehension numbers in high crime areas of Bogotá on posters. We intended to raise the perceived likelihood of detention among potential delinquents and change their ensuing behavior. The study was run as a randomized trial, using 154 complete housing blocks with, usually, four adjacent street segments as units of analysis–we use the Spanish term “manzana” to refer to this unit of analysis. Seventy-seven manzanas were randomly assigned to receive the treatment condition for three months: posters reporting arrest numbers in the direct vicinity, including also a reinforcing warning message and a picture of detained people. A series of different types of crime reports from the Colombian Police were tracked over the study period and a post-treatment survey was conducted to check for potential backfiring effects among the general population.

The poster campaign did not affect overall crime reports over the whole study period, but in line with apprehension risk updating theory, it reduced premeditated crimes. Premeditated crimes decreased by 24% (56 crimes) in the treatment area and by 7% (13 crimes) in the control area. Further, during the first month crimes decreased by 17% (19 crimes) in the treatment areas and did not change in the control areas. Perceptions of the general population were not significantly altered by the treatment, but security perception, trust in police and police performance perception all increased. Also, those surveyed were overwhelmingly aware of the posters (93% of the respondents in the treatment areas saw the posters vs. 3% in the control areas) and most “liked” them (67% of those who saw the posters).

The present study contributes to existing literature and policy practice in three ways. First, we use a specific and unique kind of communication. The information is narrowly targeted to the immediate vicinity of routine delinquents. We focus on police effectiveness “around *this* street block”, much in the spirit of place-based criminology [10], and use the actual arrest numbers for the mentioned vicinities as they tend to be high and thus surprising, given a common sense of impunity in Bogotá. Arrest numbers, along with a warning message, are hard to misinterpret by potential offenders. In other words, the communication on the poster is a direct suggestion to offenders to update their apprehension risk calculation. We are not aware of previous studies that have provided this kind of direct information on such an intimate scale. Also, different from earlier studies, we aim to separate the message from the messenger with the use of posters, in order to reduce confusion between the effect of the communication and the effect of the physical presence of the communicator.

Second, to our knowledge, this study is the first to test an offender-targeted information campaign in a high crime environment in the Global South. This represents a hard test environment considering that most crime goes unpunished in Colombia: the probability of conviction is less than 5% for thefts and homicides [11], which leads to a pervasive perception of impunity mainly associated with an inefficient judiciary apparatus [12]. While Bogotá has seen a sustained process of homicidal violence reduction over the past two decades [13], it still deals with high levels of common delinquency [14,15] and widespread perceptions of insecurity [16]. Crime prevention innovation is thus urgently needed in Colombia and Latin America more broadly, but findings from North America and Europe are not necessarily transferable due to large differences in terms of state capacity and legitimacy. Scope conditions of existing theories remain hence poorly understood. Local authorities in the city of Bogotá, and also Medellín, have recognized the need to produce locally based crime prevention knowledge and have supported a series of studies over the past years. Recent and current studies in these two cities include, for example, hot spots policing interventions in Medellín and Bogotá [17,18]; the effect of surveillance cameras and infrastructure projects on crime in Medellín [19,20]; and the effect of a survey-embedded information campaign targeted at the general public on security perceptions in Bogotá [21].

Third and with respect to policy practice, this poster campaign is an inexpensive low-tech tool that can be easily adapted and implemented in other contexts. Given the results of this experimental study, it might be used to accompany the initial stages of a broader strategy to regain control over high crime neighborhoods. Also, it might be effective in areas with large numbers of premeditated crimes, like shoplifting, thefts and illegal drug-selling. The general audience in Latin America seems to be open for such campaigns, but the approach taken in this paper by no means implies that crime prevention should not also and predominantly focus on broader social and economic dynamics that drive high levels of crime in developing countries [22].

## Information provision, deterrence and crime

We argue that information on police effectiveness can affect offenders’ risk perception and deter them from committing crime. Before we further specify this proposition, we lay out its main components, starting with deterrence and crime prevention. In Nagin’s recent review of the crime deterrence literature [1,23], he writes that the deterrent effect is mainly driven by the certainty of punishment, and not the severity, and that the “certainty deterrent effect pertains almost exclusively to apprehension probability” [1]. In turn, the perceived risk of apprehension depends mostly on the visibility and presence of the police as capable guardian. This is in line with situational crime prevention which intends to tackle the proximal causes of crime related to opportunity rather than the offender’s disposition [24].

The visibility of the police and its deterrence capacity has found support in a series of experiments on hot spots policing, starting with Sherman and Weisburd’s [25,26] seminal study of police patrol dosage in Minneapolis’ hot spots. A recent meta-analysis of Braga et al. [2] on hot spots policing largely confirms this early finding. Beyond direct police presence, the use of surveillance cameras as surrogates of police, often in company of signs indicating their presence, has also had a detectable crime reducing effect [27,28], likely through the same channel of increased apprehension risk perception.

One could argue that police officers by their very presence communicate increased risk of apprehension and thereby deter criminals from committing crimes. The question tackled in the present study is whether the information conveyed through police presence can be conveyed in other ways. Cullen and Pratt [29] ask precisely “whether the police can increase their salience—to make offenders think about them and the associated apprehension risks—even when they are not present”, to establish a kind of “virtual supervision” [30]. This reminds us of Zimring and Hawkins’ [3] initial quote, stating that the deterrence threat is a form of advertising. In line with this reasoning, Kennedy [9] also argues that “deterrence rests on the information available to and beliefs of offenders, not on the actual objective environment.”

Existing empirical evidence suggests that exposure to information can have a crime preventing effect. Smith et al. [6] identify an “anticipatory benefit of crime prevention” when they observe a crime reducing effect of the announcements that precede crime prevention activities. They interpret this finding as indicative for the role of perception: “Insofar as crime prevention reduction tactics work at all, they work through changes in perception” [6]. Other studies using different types of communication to target offenders have found similar results. Kennedy et al. [7] find that meetings with youth gang members, during the Boston Gun Project, focused on the consequences of offending resulted in measurable declines of violence. Pickett et al. [4] show that for the case of white-collar crime, sanction risk communication impacts the study participants’ willingness to commit a crime. Nettle et al. [8] find that a sign of watching eyes together with a verbal message reduces bicycle theft. Bowers and Johnson [31] identify large potential for publicity campaigns through reviewing a series of studies about, what they term, “publicity for preventive purposes,” including both offender-targeted publicity and publicity for crime victims and the wider public, mostly geared at reducing opportunities for crime [32–34].

While there is initial theoretical and empirical support for a link between offender-targeted advertising and crime prevention, several aspects of this link are not well established and we thus have to rely on a series of assumptions. First, we have to assume that information about apprehension risk, punishment severity, and other deterrence-related aspects is not fully transparent and available for neither the general public nor delinquents [9,35,36], even though some criminals are well-informed about their odds of being arrested [37]. This assumption is important since information provision can only work if the information that is provided has some novelty value for the targeted audience. If criminals were perfectly informed, an information campaign could not produce any change.

Second, existing studies theorize that the mechanism through which information works is the adjustment of the delinquents’ calculus, which most studies do not observe directly. Exceptions include Pickett et al. and Schulz who directly study sanction risk updating [4,38]. For our study, one observable implication of this suggested mechanism is that risk updating should only work for crimes that imply a minimal level of premeditation but not crimes that are carried out spontaneously.

Third, while we assume that the main channel at work is a changed apprehension risk perception of offenders, we cannot discard the influence of other channels at play. Most importantly, messages targeted at offenders might have side-effects on other citizens, for example increasing fear of crime [31,32]. While neither positive nor negative effects on citizens’ attitudes have been identified for classical hot spots policing [39–41], interventions based on publicity have exerted more positive effects. An intervention reporting objective crime trends embedded in a survey experiment in Bogotá momentarily improved perceptions of safety and police effectiveness [21], and a leaflet campaign in London had a positive impact on trust in police [42]. A change in broader citizen perception may have downstream effects on crime, for example through changed reporting practices or withdrawal from public life.

## Research strategy

### Treatment

The treatment consisted of a poster campaign. Posters were clearly attributed to the Colombian Police and contained the messaging: “Think twice. In the past two years, thanks to the community, the police have arrested XX people around this manzana. Delinquent, you will be next” (S1 Fig). In Spanish, manzanas are full housing blocks, typically including four adjacent street segments. The number of arrests were obtained from police operation reports on the level of the “cuadrante^”^. Cuadrantes are the smallest unit of police administration in Bogotá, containing between two and about 100 manzanas depending largely on the level of delinquent activity. Posters reported the actual number of arrests in each cuadrante, which ranged from 44 to 439.

The poster campaign was gradually implemented during ten days until the whole treatment area was covered, and then sustained for three months (from August 18 to November 18, 2016). Sixteen posters per “manzana” (a complete manzana usually including four sides) were initially installed by previously trained policemen. Policemen were plain clothed during the installation of posters and operated at times of the day with little traffic volume. Also, the treatment period only started once all the posters were installed. These precautionary measures were put in place to limit potential confounding between the message (the posters) and the physical presence of the messenger. As each manzana has a different structure, we abstained from using a uniform distribution scheme of posters across manzanas and rather opted for hanging posters in the most visible spots. The policemen were thus instructed to hang them for example next to shops or cash machines and at street corners. During the last two months of the treatment period, two plain clothed policemen checked three times whether posters were still in place and reposted damaged or removed posters in order to maintain the treatment for the whole treatment period (no such control rounds were executed during the first month of the treatment period). Examples of the approximately 1000 photographs they took in both treatment and control areas (to monitor their work and maintain symmetry between control and treatment areas) are included in S1 Fig. Police activity maintained its usual routine in order to not violate the excludability assumption for experimental designs [43]. In fact, police officers in the study areas were deliberately not informed about the treatment conditions.

We used posters as the medium of communication for several reasons. First, we intended to design a communication campaign that is not easily confounded with the human interaction that often comes along with communication. A leaflet campaign, one possible alternative, usually relies on distributing personnel and would have only had an effect during certain hours of the day. Although posters need to be installed by a workforce, they can then be left alone and possible effects are thus separable from the messenger. Second, since a large share of Bogotá’s crime is committed by unknown petty delinquents in public space, our treatment aimed at sending a message to this specific population, meaning that direct communication with offenders or gang members like in the Boston Gun Project was not possible [7]. Third, posters are a flexible, low-tech, and cheap solution, ideal for a test run. Later uses of deterrence advertising could include more expensive billboards or electronic news tickers. One potential problem arising with the use of posters is vandalism. Damaged posters might produce a sense of disorder and thereby contribute to crime [44]. We collaborated with the Secretary of Environment of the Mayor’s office of Bogotá to place posters in a responsible manner limiting visual contamination.

With respect to the message on the poster, we tested different versions with local experts in psychology and criminology. While we tried to reduce the poster content to an easily and clearly transmittable message, we included the following aspects: the logo of the National Police as sender of the message, the actual number of apprehension in each cuadrante as the main message visible in the center of the poster, a photograph of arrested people to reinforce the message, and a general warning message, highlighted in red, that emphasizes the target of the poster communication (“delinquent, you will be next”). In order to communicate apprehension risk, we used the one piece of information that might be considered most direct in this regard: the number of arrests [32]. This is why we excluded other high crime areas with low arrest numbers. Reporting a low arrest number in a high crime area might have produced a contrary effect. As a potential alternative, we deemed clearance rates (crimes “cleared” as a proportion of total crimes) to be more difficult to communicate and potentially misleading.

While the information conveyed is evidently limited, we argue that it should make a difference for delinquents frequenting the treatment areas. The arrest numbers reported on the posters were surprising for residents, as became clear on field visits. We thus assume that delinquents received a new piece of information that could be integrated into their decision-making process. Also, we targeted information to the specific area (“around this street block”) in order to increase the perception of police effectiveness in the viewer’s very location, rather than on a more aggregate and abstract level [4,31,37]. A small proportion of delinquents in our study areas may have been unable to read and write. According to the National Prison Institute (INPEC), about 5% of detained people are analphabets. A similar share of illiterates can be expected in our study areas. While the treatment may be less effective for this subgroup, we included a visual component (picture of arrested people) to appeal to illiterates.

Somewhat similar communication strategies have already been used around the world. In London, posters with a pair of eyes and a warning notice were used [45]; in Boston, cardboard cutout cops were used to deter bike thieves [46]; and the Singapore Police uses electronic billboards for both informing communities and deterring criminals [47]. However, robust research on the impact of such interventions has been scarce to our knowledge, even in a Northern context. Also, interventions used in one context do not necessarily travel to another, especially given the very specific local conditions in Bogotá. A tailor-made approach for common delinquency in South America, as proposed here, thus requires separate testing.

### Unit of analysis, selection of manzanas and randomization

Since it is logistically impossible to cover all Bogotá and since we are interested in areas with the highest return on investment, we focus on high crime areas. Crime in general (47), and in Bogotá [48], usually occurs in a small number of street segments [49]. Also, the deterrence threat of our poster campaign can only be credible in areas with large arrest numbers. As unit of analysis, we focus on manzanas (full housing blocks with typically four adjacent streets). Previous research has focused on street segments (one block) or street corners. We use the slightly larger manzanas in order to increase the likelihood of some crime occurring during our relatively short treatment period of three months. Prior conversations with the Police led to the conclusion that 75 manzanas was a manageable size for the poster treatment. In order to generate a total of around 150 initially targeted manzanas, we first selected around 1000 manzanas that reached a threshold of at least 10 registered crimes or that had a high amount of emergency calls due to either theft or personal attack during the year 2014 (see section on Data).

We then excluded manzanas from the initial pool based on the following criteria: first, we selected only one manzana per cuadrante (the one with the highest crime level), since we reported information on posters on the cuadrante level. Second, we excluded directly neighboring manzanas from adjacent cuadrantes, in order to avoid interference between treatment and control. Third, we excluded manzanas with very low reports of arrests despite high crime or emergency call numbers. Fourth, we excluded few additional manzanas for different reasons: for example the central police station that registers crime from all over the city; manzanas with evident mistakes in geo-referenced data; and manzanas that risked raising unnecessary public attention for the study (to our knowledge, the poster campaign was not noted by the media). In conclusion, we were left with 154 manzanas with a wide geographical spread across the city that entered the final study and were randomly divided into a treatment group (N = 77) and a control group (N = 77). For illustrative purposes, we include an example of an area with several selected manzanas and photographs of the four corners of one selected manzana in S2 Fig. All selected manzanas were inspected before the treatment began.

The number of manzanas was largely determined by logistical constraints. Since there are no comparable studies to date that would allow for a specific effect expectation, we provide power calculations for a range of expectations with regard to the treatment period. Based on comparison data from 2015 (three months total crime in our 154 manzanas), we calculated the power for a sample of 154 manzanas at 19.9% for detecting average reported estimates of regular hot spots policing effects (0.18 standard deviations) and 99.9% for high estimates of hot spots policing effects (0.87 standard deviations), as reported in Braga et al. [2]. The magnitude of effect needed for a P = .05 difference to have an 80% chance of detection with the 154 manzanas for the difference between treatment period and a comparison period in the preceding three months corresponds to 1.19 less crimes per manzana (or 0.45 standard deviations).

Assignment to treatment was carried out in three stages using complete random assignment [43]. First, manzanas were ranked from highest to lowest on total registered crimes during 2014/5. Second, pairs of manzanas with similar levels of crime were generated. Third, from each pair, one manzana was randomly assigned to the treatment and one to the control group. With this strategy, we intended to avoid assigning a small number of manzanas with very high levels of crime to the same group.

Since the start of the treatment was postponed several times, the data used for the initial randomization (from 2014/5) does not coincide with the pre-treatment periods we use in this study. While the control and treatment areas are balanced on most pre-treatment indicators (S1 Table), the control manzanas have consistently lower levels of overall crime than the treatment group. As defined in the pre-analysis plan, the analysis is conducted with difference variables (treatment period minus pre-treatment period), which accounts for this baseline difference, and covariates.

### Hypotheses

The main objective of this study was to evaluate the crime reduction potential of our poster treatment. Our first pre-registered hypothesis was thus straightforward:

Hypothesis 1: Manzanas in the treatment group will have a reduced number of crimes during the study period as compared to manzanas in the control group.

We also argued that the effect of our treatment would be strong for premeditated crimes (e.g. thefts, drug dealing, gun possession) and less strong or absent for impulsive crimes (e.g. personal injuries) as the deterrence threat targets the decision-making process of routine criminals rather than crimes committed in the spur of the moment [50,51].

Hypothesis 2: The reduction of crime will be larger for “premeditated” crimes than for “spontaneous” crimes.
In order to identify temporal aspects of our treatment effects, we propose an additional hypothesis grounded in existing research on police crackdowns and anticipatory benefits of crime prevention [6,52]. Sherman [52] argues that offenders learn through trial and error and recognize that they overestimate the certainty of getting caught after an initial period. That is why we expect a larger effect in the initial phase of the treatment period.
Hypothesis 3: Manzanas in the treatment group will have a reduced number of crimes during the initial period of the treatment as compared to manzanas in the control group. (Hypothesis 3 was changed from the pre-registered version, which focused exclusively on the post-treatment phase. This change is also due to a change in crime reporting practices in Colombia due to the new Police code starting in 2017.)

Spatially, we expected that our intervention would show similar effects as past hot spots policing, namely not displacing crime but reducing crime in the target area and in neighboring areas through a diffusion of benefits [53]. This expectation is based partly on a recent meta-analysis by Johnson et al. [54] who find that crime in wider catchment areas of policing interventions remains the same or decreases slightly.

Hypothesis 4: Neighboring areas of manzanas in the treatment group will have a reduced number of crimes during the study period as compared to neighboring areas of manzanas in the control group.

While our intervention is targeting offenders, citizens living and working in the respective neighborhoods might unintentionally be affected by the poster campaign [31]. The direction of this effect is though debated in the literature. Specifically, an offender-targeted communication campaign might produce a negative backfiring effect on a broader audience. However, existing studies do not identify such an effect for similar interventions, like hot spots policing, or even find a positive effect [39,42]. We thus operate with a positively formulated hypothesis, although the empirical priors are not clear.

Hypothesis 5: Residents and workers of manzanas in the treatment group will improve their level of trust in the police, the perceived police effectiveness and their security perception.

### Crime data from the police

Most data for this study is provided by the Colombian National Police. Data was extracted for a *pre-treatment period* (May 18 to August 17, 2016–92 days prior to treatment) and a *treatment period* (August 18 to November 18–92 days) for both manzanas in treatment and control areas. The police officer in charge of extracting the data was blinded with regard to the treatment status of the studied areas.

For hypothesis 1, we use the totals of registered crime, emergency calls for theft and attack, and minor wrongdoings in the 154 control and treatment manzanas. The Police registers crimes according to penal code entries. During the pre-treatment period, personal theft (262 events), shop theft (67) and personal injury (64) were the most common of the 606 registered crimes in the 154 manzanas (full list of crimes in S3 File). Total registered crimes are the primary outcome measure for our analysis. Emergency calls to the main emergency call line in Bogotá (phone number 123) are coded by call receivers with a standard format. We use theft (510 events in the pre-treatment period) and attacks/street fights (in Spanish “riñas”– 1840 events) as key categories for our study. Total calls for theft and attack are a secondary outcome measure, they allow us to account for potential problems of crime registration. The police also registers minor infractions which are not directly sanctioned, but require an admonition by the police. Minor wrongdoings (511 events in the pre-treatment period) include urinating in public space, threatening neighbors, making excessive noise, etc. (full list in S3 File). We use these public order disturbances as additional secondary outcome measure for our analysis.

For hypothesis 2, we construct separate variables for premeditated and spontaneous crimes based on the crimes registered by the Police. Premeditated crimes include (as in the pre-registered study design) personal, commercial, motorcycle, residential and car thefts, illegal drug possession and dealing, and illegal gun possession and dealing. Spontaneous crimes include personal injury, unintentional injury, domestic violence, and sexual abuse. While these indicators do not represent a perfect operationalization of the level of premeditation of a crime, they do represent a general indication and also include the three most common crimes in Colombia (theft, drug crimes, and personal injury). For the operationalization of this variable we consulted officers working at the Criminal Investigation Unit of the Colombian Police (DIJIN). We also use emergency calls for thefts as rough indicator for premeditated and calls for attacks as rough indicator for spontaneous crime.

For hypothesis 3, separate panel day to day datasets for total registered crime were created to account for the temporal evolution for different periods of the treatment: first, second, and third month. We abstained from using a series of additional cut-off points to slice the three months in shorter periods, partially because crime in manzanas is a rare occurrence and under-aggregation might produce noisy measurements.

For hypothesis 4 about neighboring areas, data on registered crime, emergency calls, and minor wrongdoings was extracted for the whole “cuadrante” excluding the specific targeted manzana in order to identify crime in directly adjacent areas. We use the cuadrante level as catchment area for two reasons. First, using the cuadrante level, instead of a constant catchment radius around the study manzanas, allows for avoiding overlapping catchment areas, as neighboring cuadrantes may contain treated or control manzanas as well. Second, as our study treatment refers to the cuadrante level (arrested people reported on the posters are drawn from that level), we opt for using this same cuadrante as wider catchment area for measuring potential nearby crime displacement.

### Survey data

For hypothesis 5, we use survey data from residents and workers of the treatment and control areas. We conducted a post-treatment survey only, due to budget constraints. Given the high crime context, the survey was conducted by junior police officers (most of these officers were blinded with regard to the experimental condition). The enumerators wearing plainclothes were identified as working for a research project at Universidad de los Andes in Bogotá and were thus not recognizable to the participants as members of the police. The survey was executed during the first three weeks after the treatment (November 22 to December 12, 2016). After receiving informed oral consent, enumerators asked questions about demographic characteristics, crime victimization, security perception and trust levels in different institutions for no more than 10 minutes (full original survey in S1 File).

Due to the diverse nature of the manzanas (mainly residential and commercial) and the informal characteristics of some of the areas, we abstained from constructing a sampling frame and relied on a quota sampling procedure, which was carried out identically for treatment and control areas, thus guaranteeing a symmetrical measurement. We interviewed four persons in each manzana (two below 35 years of age and two above, two female and two male respectively) for a total sample size of 616 individuals. Residents and workers of the respective neighborhoods who had a realistic chance to see the posters in the preceding three months were selected for the survey. Occasional passers-by were excluded. Demographic characteristics of the surveyees in control and treatment areas are largely balanced, except for education level and media consumption (S1 Table).

The survey confirms the high vulnerability of people living and working in the studied areas and a generally distrusting relationship with authorities: 64% of the surveyed population stated that they were direct victims of crime in the last three months while 34% of them reported the crime to the police. The survey also shows whether study participants were aware of the treatment. While the surveyed people were likely not the main audience of our treatment (we were interested in changing the delinquents’ calculus), their awareness of the poster campaign is an important indication of its visibility. Of the people in the treatment group, 93% recalled having seen the posters vs. 3% in the control group (these proportions remain identical if we exclude surveys conducted by non-blinded enumerators). This indicates a high visibility of the treatment among the general population, likely also among the delinquent population of the treated areas, and a low risk of interference between treatment and control areas. Of those who saw the posters, 67% liked it, 4% disliked it and 28% were indifferent about them, showing that the poster campaign was generally welcome.

## Findings

We report results according to the outlined hypotheses using a difference in difference approach to account for pre-treatment differences. The main outcome measures thus represent the average manzana level difference between the treatment and pre-treatment period. In accordance with our pre-analysis plan, we analyze data with linear regressions and simple t-tests. Instead of showing a series of tables, we present the results with coefficient plots to facilitate reading (for full regression and t-test tables, see S2 Table). The coefficients are drawn from linear regressions which include a set of covariates. For hypotheses 1 to 4, the covariates include a variable that accounts for the ranking of pairs of blocks that were used for randomization, the distance of each manzana from the main central square of Bogotá to control for unequal police presence, and the amount of pedestrians (counted by plainclothes police officers during their control rounds) to control for unequal opportunities for crime. For the analysis of hypothesis 5 based on survey data, we use sex, age, residential status, education level, socio-economic strata, and media consumption as covariates. All coefficient plots show the average treatment effects on the indicated outcome measures, using 90% and 95% confidence intervals. Intervals that do not overlap with 0 indicate a significant difference between treatment and control group. For hypothesis 1 to 4, coefficients below 0 indicate a crime reduction in the treatment group compared to the control group.

### Hypothesis 1

**Fig 1:**
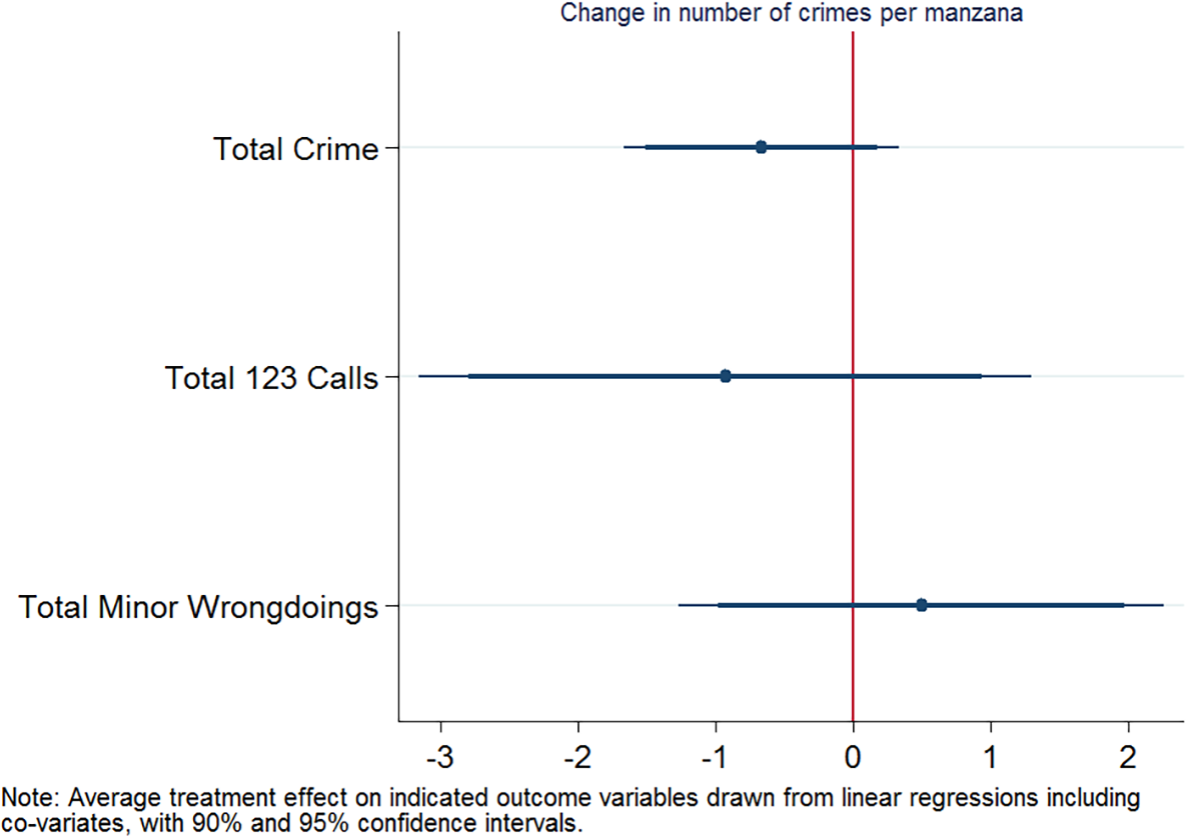
Coefficient plot for hypothesis 1.

For hypothesis 1 (in short: reduced overall number of crimes in treatment vs. control manzanas), we report the average treatment effects on total registered crime, total calls to the emergency line for thefts and attacks, and total minor wrongdoings over the whole treatment period of three months in Fig 1. While total registered crime and calls to the emergency line were more reduced in treatment than control areas, differences are not statistically significant for any of the three main outcome variables.

### Hypothesis 2

**Fig 2:**
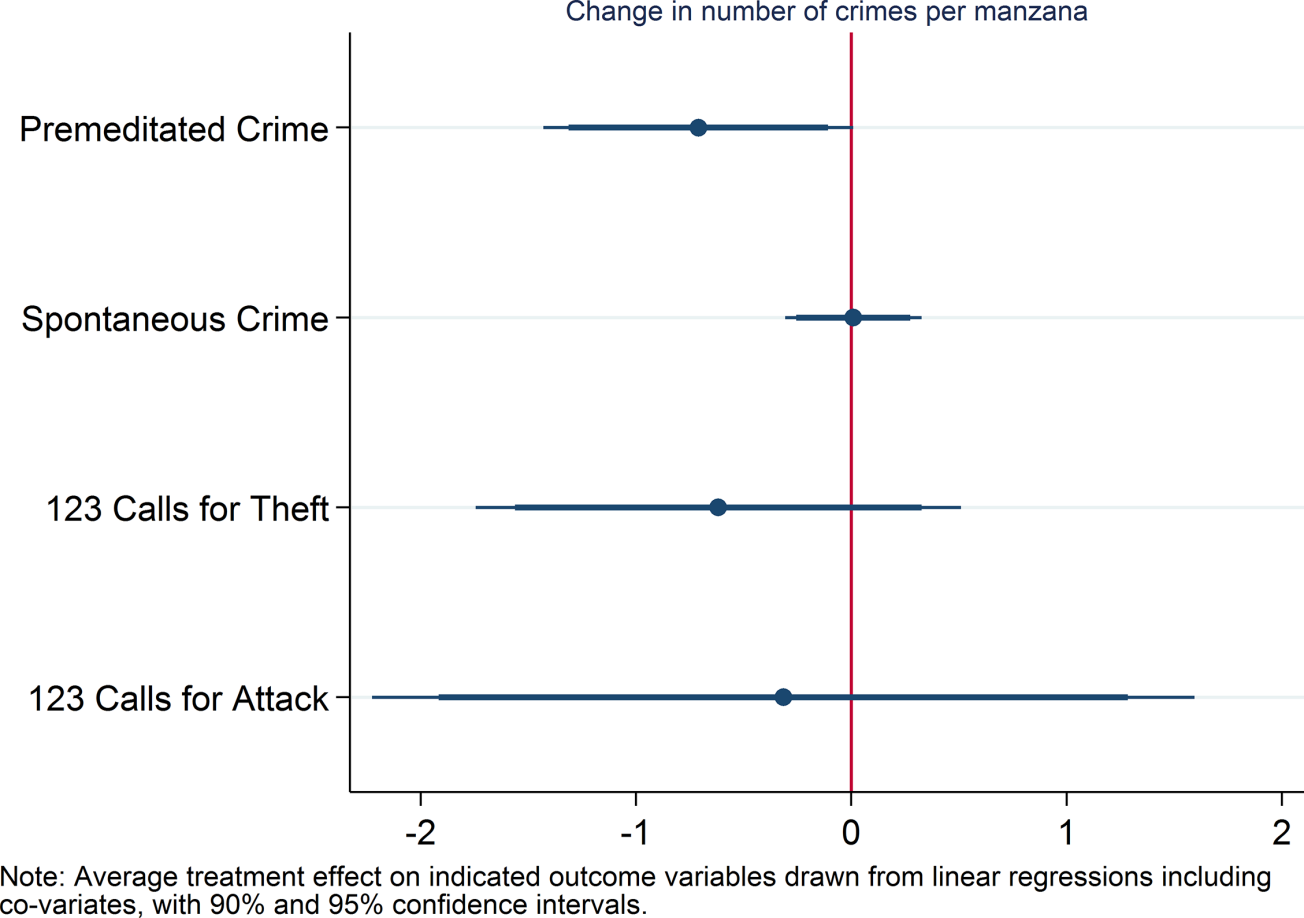
Coefficient plot for hypothesis 2.

For hypothesis 2 (larger reduction for premeditated than for spontaneous crimes), we report the average treatment effects on reports of the above defined premeditated and spontaneous crimes, and on emergency calls for theft and attack over the whole treatment period. As indicated in Fig 2, premeditated crimes are significantly more reduced in treatment than control areas (the t-test reported in S2 Table also indicates a p-value of .05), while spontaneous crimes are the same for control and treatment areas. Premeditated crimes decreased from 235 to 179 (24% reduction) in treatment areas and from 175 to 162 (7% reduction) in control areas. The secondary indicators for this hypothesis (theft and attack) show no statistically significant differences, but the substantive effect is stronger on thefts (generally premeditated) than attacks (predominantly spontaneous).

### Hypothesis 3

**Fig 3:**
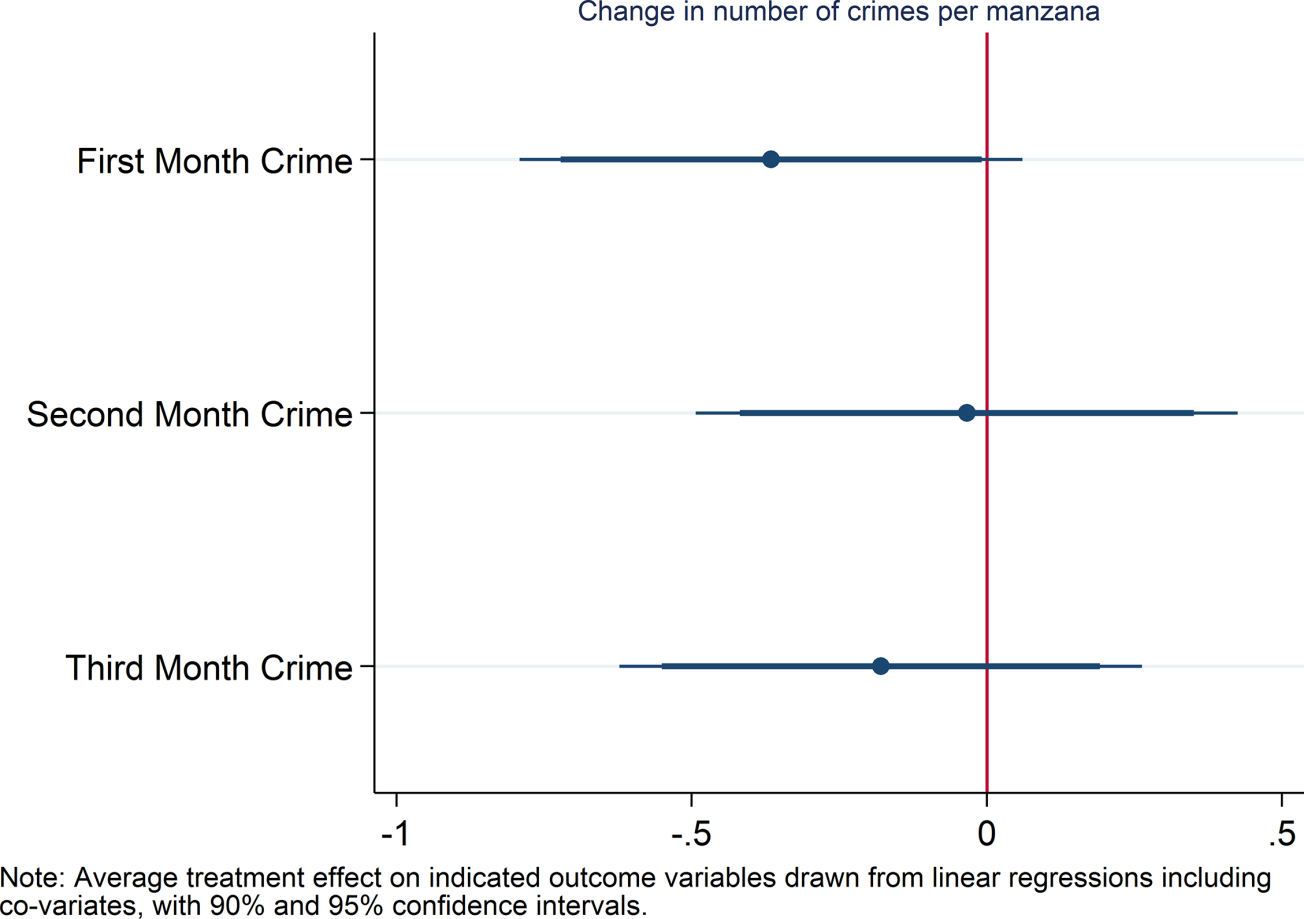
Coefficient plot for hypothesis 3.

For hypothesis 3 (reduced number of crimes in treatment vs. control manzanas during the initial period), we distinguish between different phases during the treatment period. We use the difference between treatment and control areas for the first, second and third month respectively and the pre-treatment period (see Fig 3). For the first month, we find an average treatment effect significant at the 10% level, but not for the second and third month (this result is statistically weaker in a one-sided t-test without co-variates, see S2 TAble). Registered crimes decreased from 113 per month in the pre-treatment period to 94 in the first month of treatment (17% reduction) in treatment areas and stayed the same in control areas.

**Fig 4:**
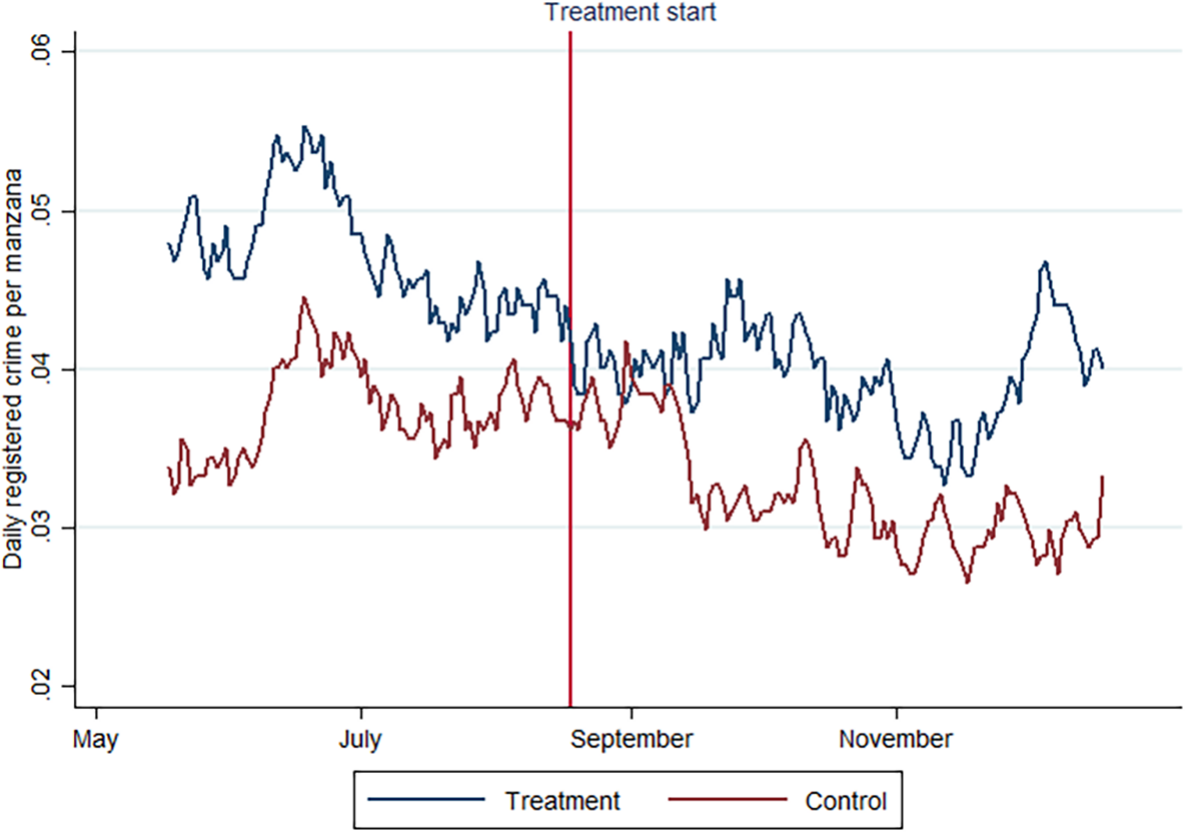
Ten day moving average crime in treatment and control areas.

A temporal effect is visible in a relative dip in registered crime in the treatment areas right after the treatment started, as shown in Fig 4 (the red vertical line indicates treatment start). While there seems to be a baseline difference between crime in control and treatment areas (with treatment areas having a generally higher level of crime), this difference disappears for the first month of the treatment. The graph shows ten day moving averages for registered crime in treatment and control areas. We selected ten day moving averages for visualization purposes. Considering that the treatment was gradually implemented during ten days before August 18, the treatment start cannot be seen as clear cut and a moving average representation is thus appropriate.

### Hypothesis 4

**Fig 5:**
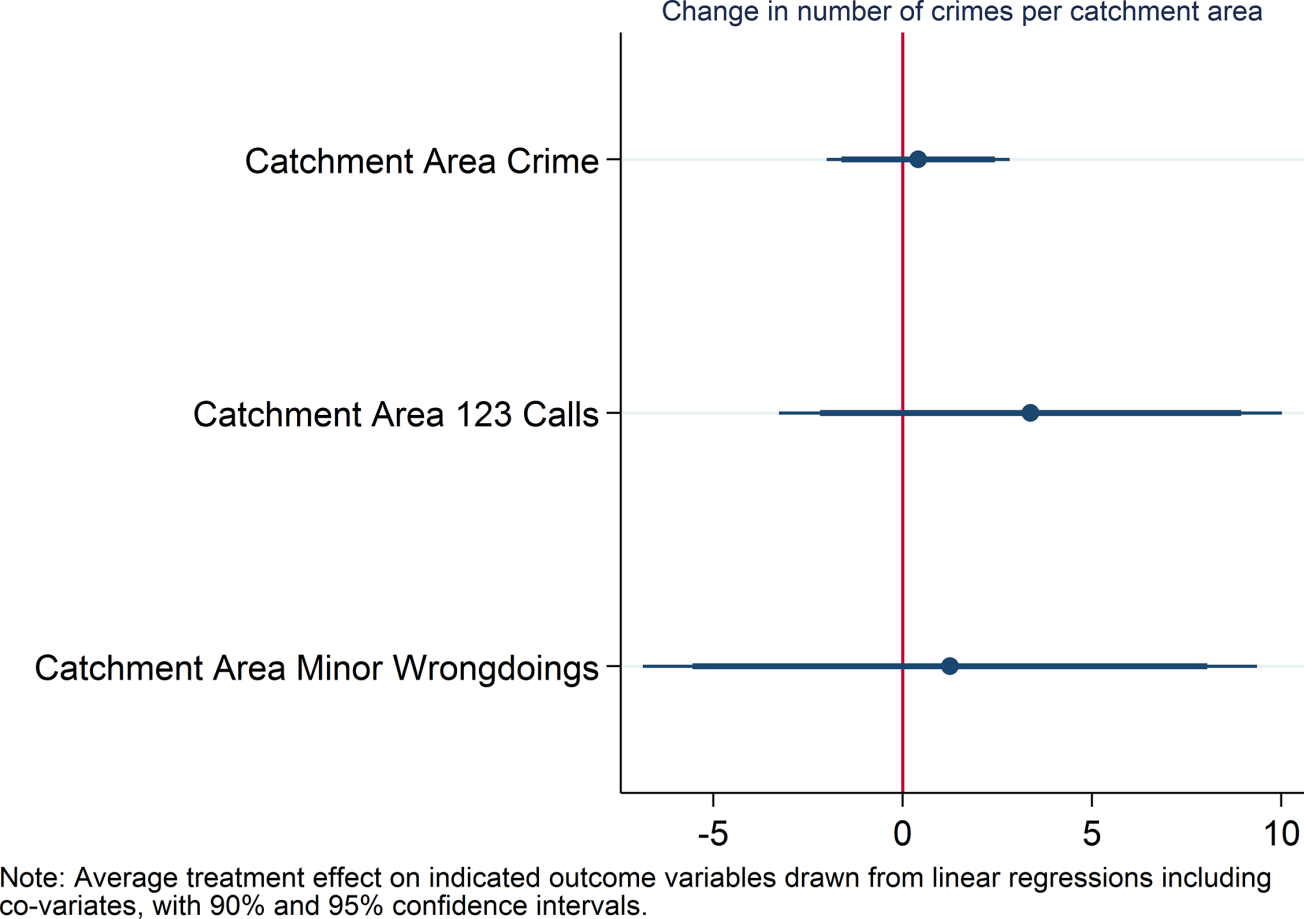
Coefficient plot for hypothesis 4.

For hypothesis 4 (reduced number of crimes in neighboring areas of treatment vs. control manzanas during), we focus on the levels of crime in the wider catchment area around the treated manzana (the neighboring areas), using the same outcome measures as for hypothesis 1. All three indicators are positively related with the treatment. If anything, the treatment has an effect of displacing crime into adjacent areas rather than a diffusion of benefits effect. However, Fig 5 shows that there are no statistically significant associations.

### Hypothesis 5

**Fig 6:**
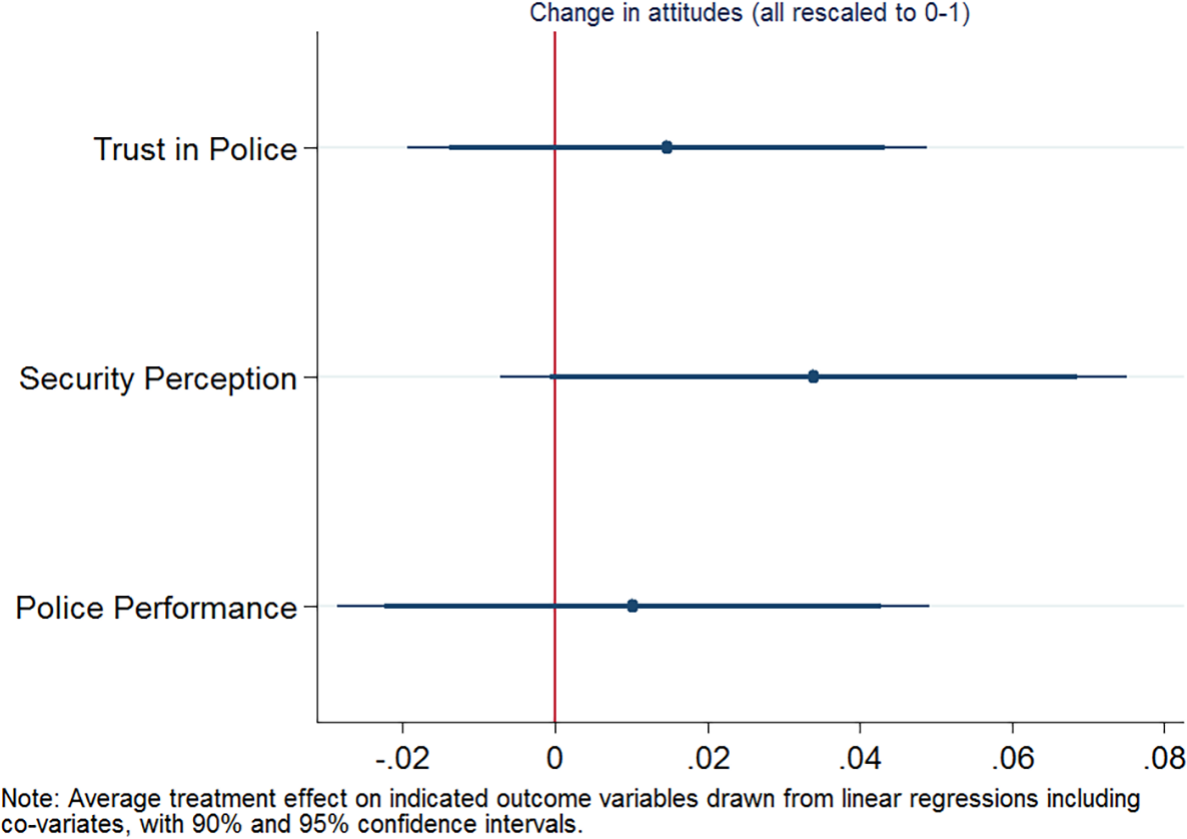
Coefficient plot for hypothesis 5.

For hypothesis 5 (improved trust in police, perceived police effectiveness and security perception in treatment vs. control manzana surveyees), we use separate data collected from a post-treatment survey. As defined in a pre-analysis plan, we use trust in police, security perception and police performance as key indicators (for their construction, see S2 File). For all three indicators, the treatment shows a positive coefficient, hence a more improved view in treatment areas than in control areas (see Fig 6). However, none are significantly related. Security perception has a p-value of .106 in the regression setup. Considering one-sided t-test results (S2 Table), both trust in police and police performance are significant at the .10 level.

## Discussion

We found that premeditated, but not spontaneous, crimes were reduced with our version of offender-targeted advertising. This is in line with the theory of apprehension risk updating. This kind of updating can only work for delinquents who do not act impulsively. It thus speaks to the same mechanism encountered in a previous study of Pickett et al. [4]. While we do not measure apprehension risk updating directly, we are confident that the posters were hard to misinterpret as they clearly recommended delinquents to recalculate apprehension risk.

Similar to earlier studies, the poster campaign was most effective during the first month but not during the second and third months. This corresponds to what Sherman (1990: 10) called the “initial deterrence decay”. The reasons for this decay are, however, not well established. It may well be that the information provided in the treatment areas loses its novelty status after a while. Also, if delinquents do not perceive any additional changes coming along with information, they may go back to their usual sources of information (their own and others’ everyday experience) and practices after a prudential waiting period.

Although the treatment did reduce premeditated crime and, to a more limited degree, overall levels of crime in the beginning, over the whole course of the treatment period, the implemented poster campaign did not significantly affect crime reports, emergency line calls and minor wrongdoings, or significantly displace crime into neighboring areas. The most important reason for a limited mid-term effectiveness may be related to the specific context of the advertising campaign. In Colombia in general, but even more in high crime areas of Bogotá, there is a pervasive sensation of a non-functioning judiciary system. Our survey exemplifies that clearly. While 51% of the surveyed people had at least some confidence that the police would arrest a delinquent if they fell victim to a crime, only 20% believed that the judiciary system would punish the delinquent, which could explain low reporting rates. Newspaper titles such as, “Thief arrested 44 times and always set free” [55] or “Arrested 63 times for theft and fraud and only sentenced to one year” [56], have contributed to this feeling of impunity. A study using interviews with criminals convicted for theft and homicide found that their perceived probability of sanction is very low even after being arrested [57]. The bottleneck of the Colombian criminal justice system is thus not so much the police but the courts. In the chain of relevant steps related to the certainty of punishment–apprehension, prosecution, conviction and sanction–the certainty of apprehension may thus be relatively less important than in a Northern context [1], given that effective sanction is unlikely. Cook [58] observes: “If active criminals find that they are rarely arrested, unlikely to be convicted if arrested, and unlikely to be sentenced to prison terms if convicted, then they may acquire a justified sense of invulnerability.”

The poster campaign may have been more effective with a different framing of the message or another channel of communication. We intentionally tried to convey isolated information on a standalone communication channel, in order to avoid a treatment that is hard to unbundle and to separate from the physical presence of a messenger. It is likely that the campaign would have been more effective with additional elements, like information about upcoming concrete crackdowns [6], or the presence of additional police forces, communicated through a leaflet campaign. Also, the offender-targeted advertising could have been complemented with elements targeted at the larger community to build better police-community relationships, which might help reduce crime in a more sustained manner [42]. The isolated nature of the poster communication make the results all the more noteworthy. A clear deficit of the poster as a communication channel is its vulnerability to vandalism. During control checks later into the treatment period (second and third month), treatment areas had on average 2.2 (out of 16 initially placed) posters, before additional posters were placed again. This might have contributed to an effect that is only detectable in the beginning of the treatment period.

Further, our treatment might have affected crime reporting practices. While the relationship was statistically weak, all three indicators for security and police perceptions point in a positive direction. If anything, the main results might thus have been tempered since reporting could have increased in treatment areas as a result of increased trust and effectiveness perception (although the relationship between trust in police and crime reporting is not settled [59]). However, the survey also included a more direct question about reporting of actual crimes in the last three months and no difference was found between treatment and control area respondents. Victimized respondents were asked: “Did you report the crime to any institution?” The percentage of yes answers was 34.95% (treatment area) and 35.7% (control area) respectively (difference: 0.75%, t = -0.15).

Scholars have raised concerns about potential backfiring effects of crime prevention publicity and similar communication interventions [31]. The survey used to analyze this question did not reveal any negative effects in terms of trust in authorities and security perception. The results might have been different if the survey had been conducted right after the start of the treatment when the posters were new not only for the offenders but also for the communities. A potential momentary change (that we could not register) was almost invisible right after the end of the treatment period. However, it is fair to deduce that the treatment did not negatively affect perceptions of the broader audience. Moreover, people in the treated areas reacted positively to the posters. Very few of those who were aware of the posters expressed displeasure (4.6%). In a study area where two thirds of the population have been victimized in the last three months, people may like almost any intervention that has potential to reduce crime.

## Conclusions

In this study, we tested an offender-targeted advertising campaign using posters in high crime areas of Bogotá. We theorized that information about high arrest numbers in an offenders’ vicinity would change their apprehension risk perception and affect their decision-making process. The campaign was sustained during three months and reduced premeditated crimes during the whole treatment period and overall crimes in the first month of the treatment period. Over the whole treatment period, it did not produce significant results for overall crime levels, emergency line calls for theft and attacks, or minor wrongdoings. Also, it did neither displace crime nor reduce crime in neighboring areas. Generally speaking, the effects were likely tempered by the hard test case environment for this study. A feeling of impunity is pervasive in Bogotá and may thus limit deterrence-based interventions. A post-treatment survey did not identify any negative side-effects of the treatment among the broader community and its views of the police and security.

Given the low cost of information campaigns (as opposed to additional physical presence of the police) and its adaptability, this kind of intervention has value for future applications. However, an indiscriminate application cannot be justified with the presented results. An information deterrence campaign as the one studied here might work best for premeditated crimes (like shoplifting, theft or robberies) and during the initial phase of broader crime prevention policies. Given that the deterrent effect of the posters may decay relatively quickly, a temporary use of this type of strategy is most adequate. As posters are vulnerable to vandalism, it may be more cost-effective to invest in physically robust communication tools, like billboards, that do not require constant maintenance.

Future studies should vary diverse aspects of the information treatment to identify its precise scope conditions and most effective use. For studies in the Global South, it may be worthwhile framing messages not only around apprehension risk but also around effective punishment, given the pervasive sense of impunity that might limit the deterrence capacity of messages focused exclusively on apprehension risk. Noting the number of police officers in the vicinity rather than arrest rates might also be effective. In a Northern context, the same treatment should generally have a larger crime reduction potential, considering the better functioning criminal justice system. Following up with effectively arrested criminals, perhaps using qualitative interviewing, might produce additional leads with regard to the information updating processes used by offenders.

## Supporting information

S1 Fig Poster treatment. (DOCX)

S2 Fig Manzana selection. (DOCX)

S1 Table Balance tables. (DOCX)

S2 Table Regression and t-test tables for hypotheses 1 to 5. (DOCX)

S1 File Full original survey Spanish and English. (DOCX)

S2 File Question framing for hypothesis 5. (DOCX)

S3 File List of crimes and minor wrongdoings. (DOCX)
