# Supplementary figures and images for: Deterring delinquents with information. Evidence from a randomized poster campaign in Bogotá
Source: PLoS One. 2018 Jul 19;13(7):e0200593. doi: 10.1371/journal.pone.0200593 (PMC6053166; doi:10.1371/journal.pone.0200593)

**S1 Fig. Poster treatment**

**Template for poster**

**
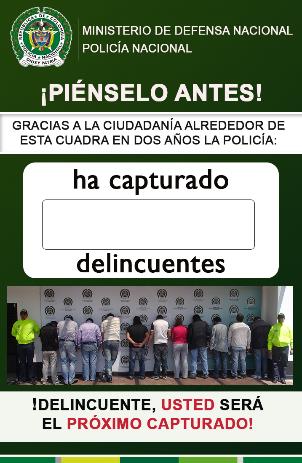
**

**Pictures of installed posters in treatment areas**


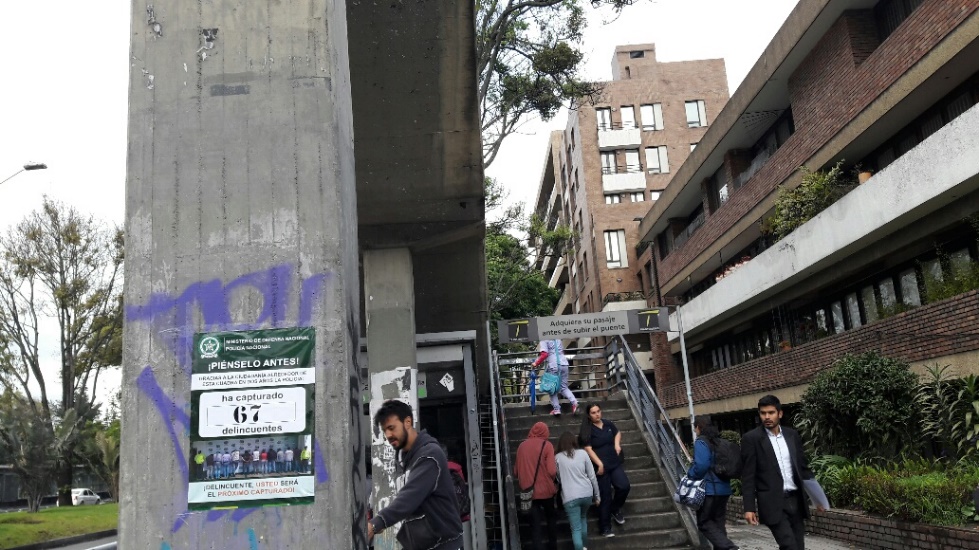


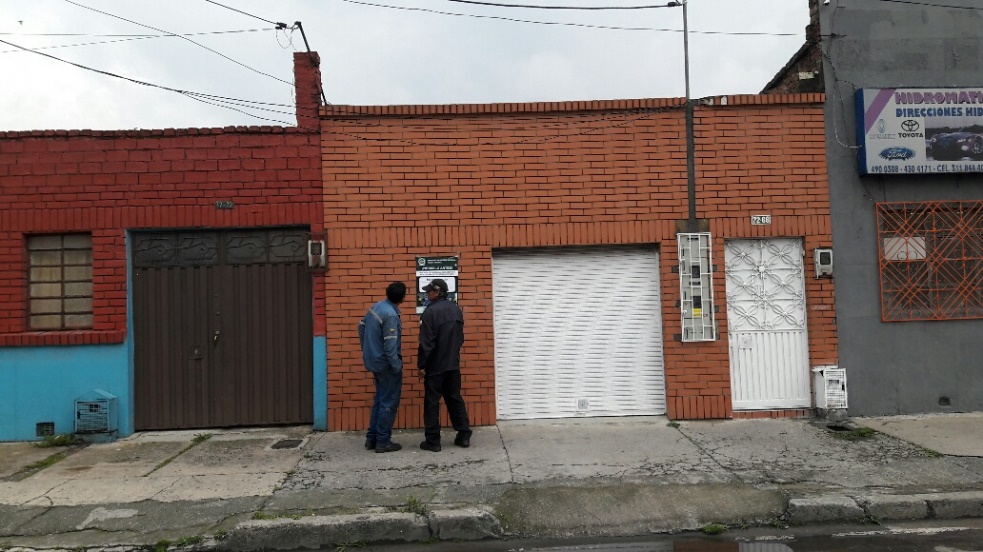


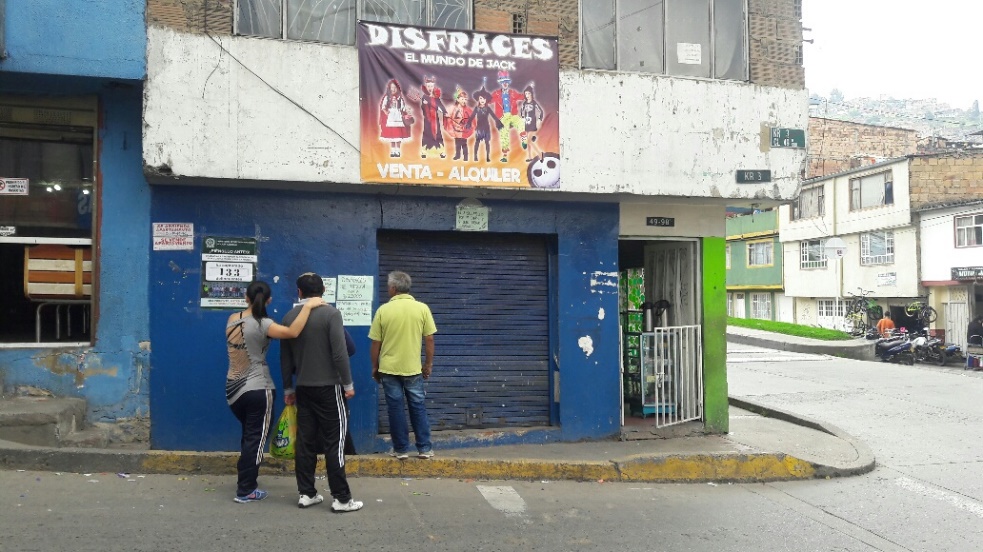


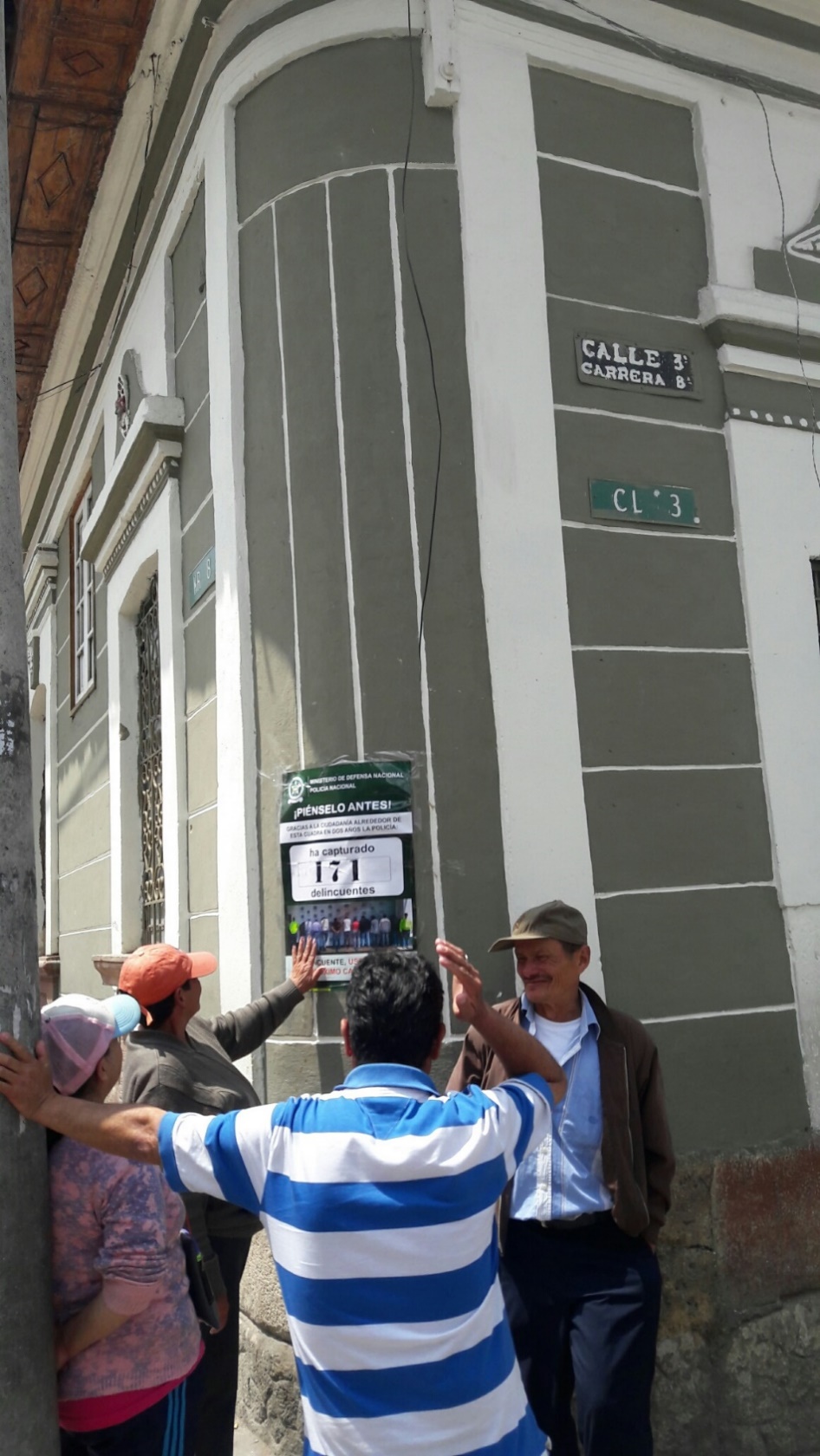


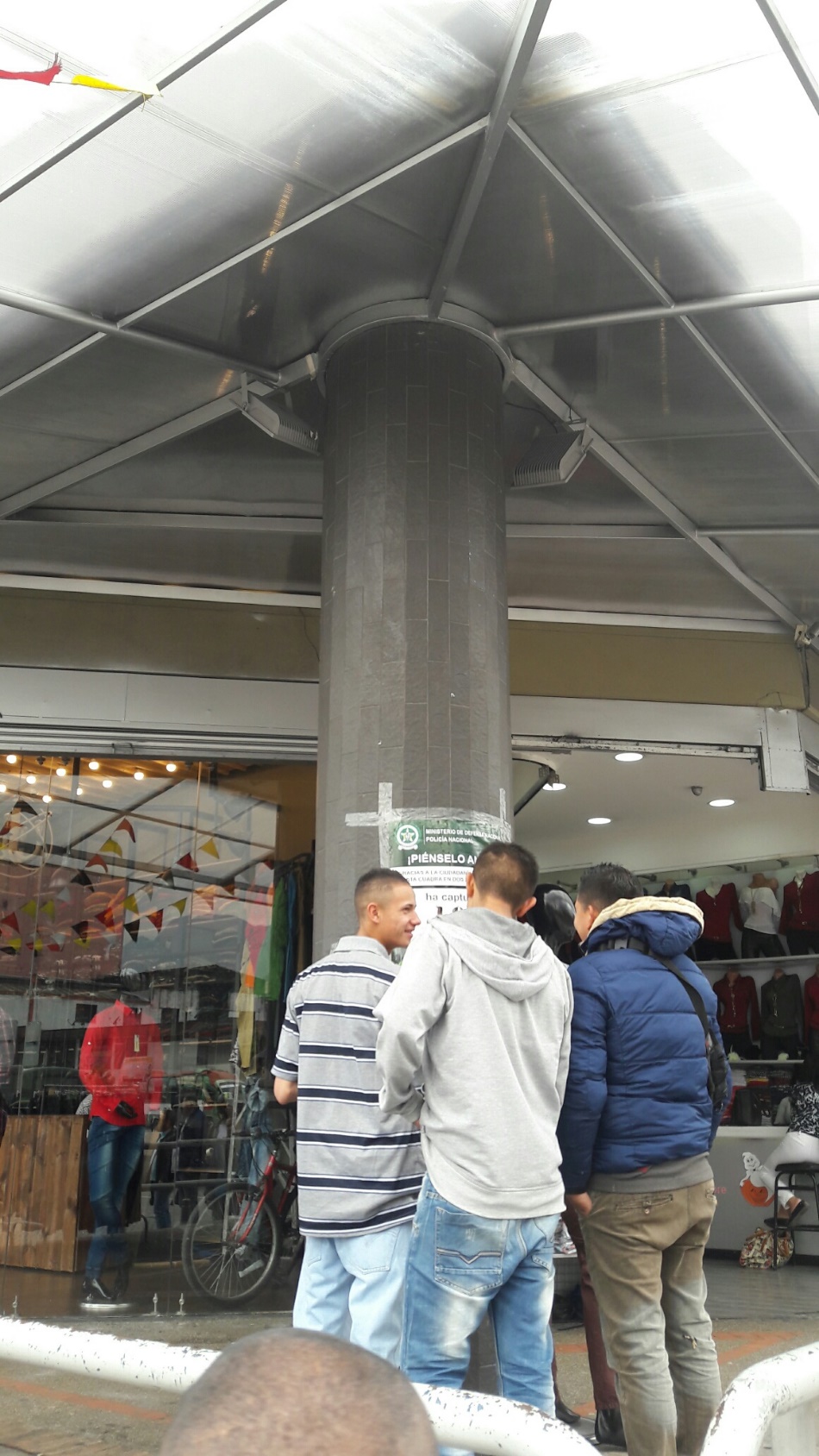


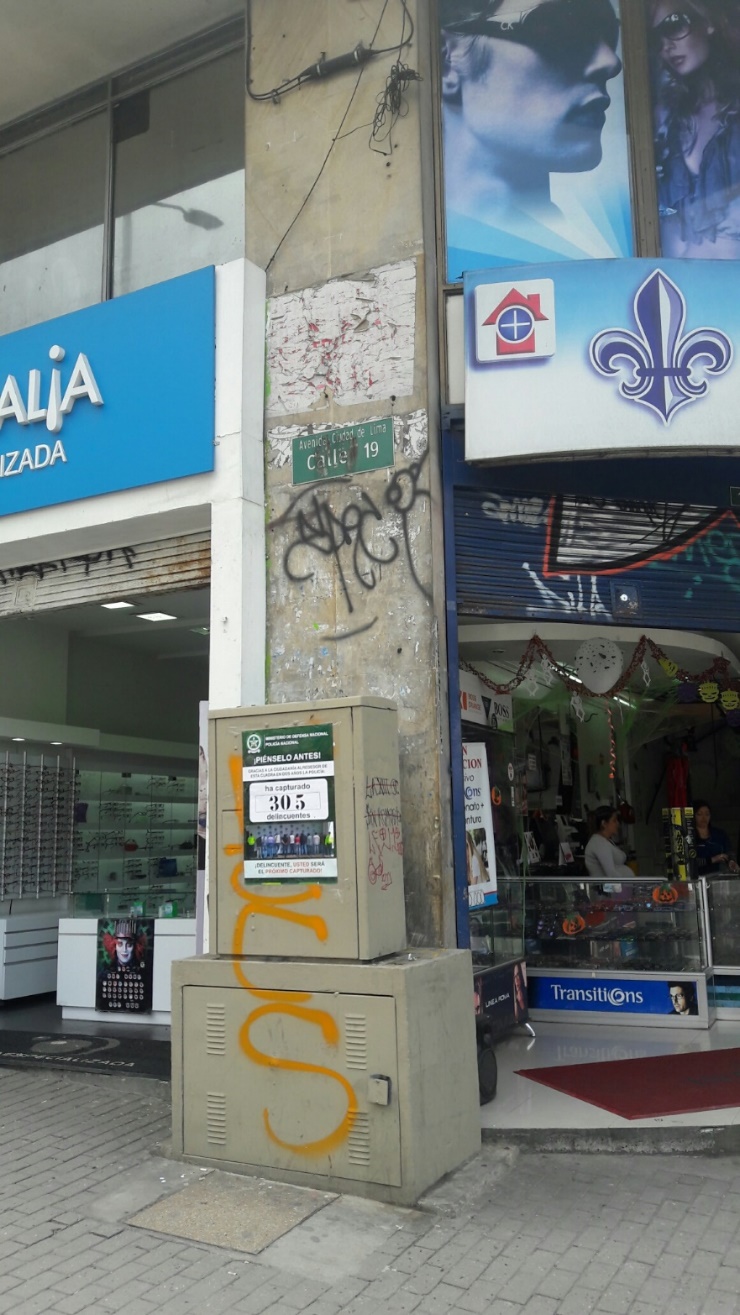


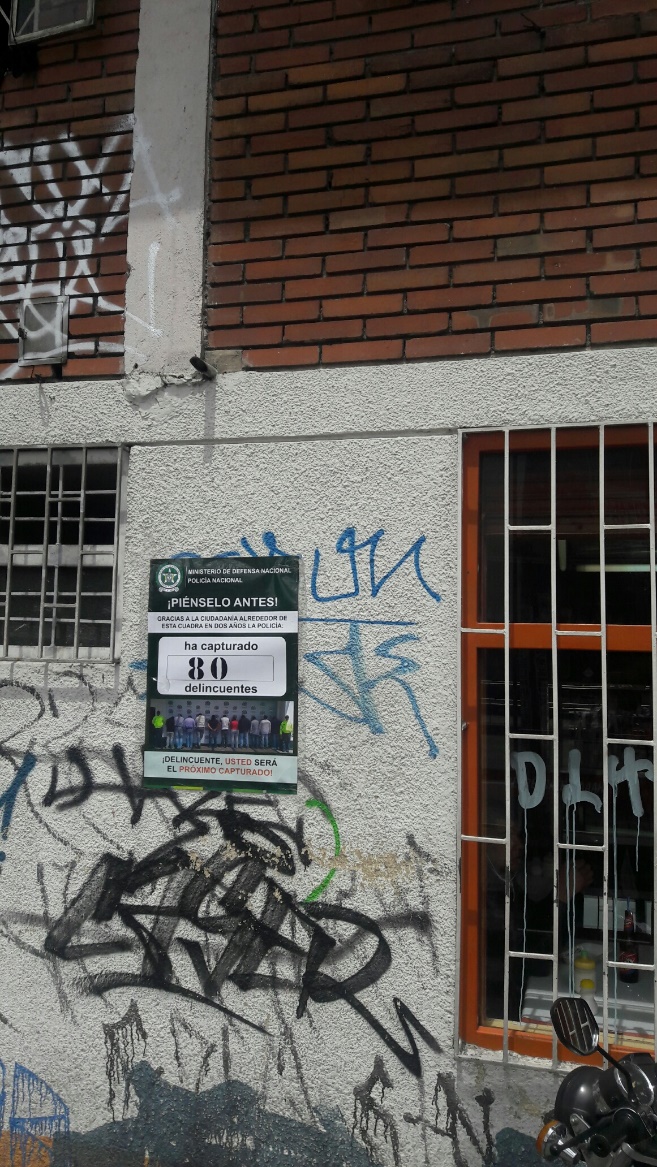

Supplement: S1 Fig — (DOCX) [file pone.0200593.s001.docx]
