# Supplementary material for: Deterring delinquents with information. Evidence from a randomized poster campaign in Bogotá
Source: PLoS One. 2018 Jul 19;13(7):e0200593. doi: 10.1371/journal.pone.0200593 (PMC6053166; doi:10.1371/journal.pone.0200593)

**S2 Fig. Manzana selection**

In the following image, blue dots represent crimes, green lines represent cuadrante limits and red shaded areas represent manzanas selected for our study. Remember that cuadrantes are the smallest police administrative areas in Bogotá that include several manzanas (depending on level of criminal activity).


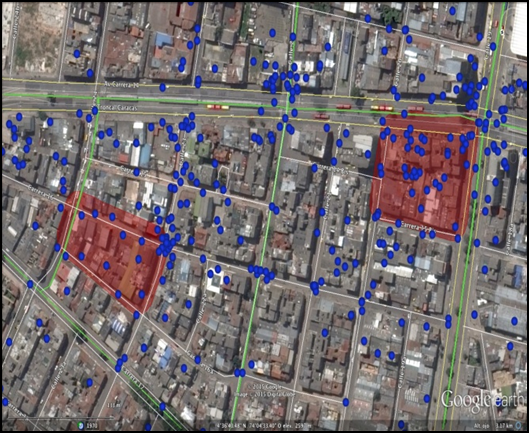


The following image is from a report of a police officer who visited all the manzanas selected for the study prior to treatment. The first two images show the full manzana in red and without red coloring. The next four images show the four corners of the manzana.


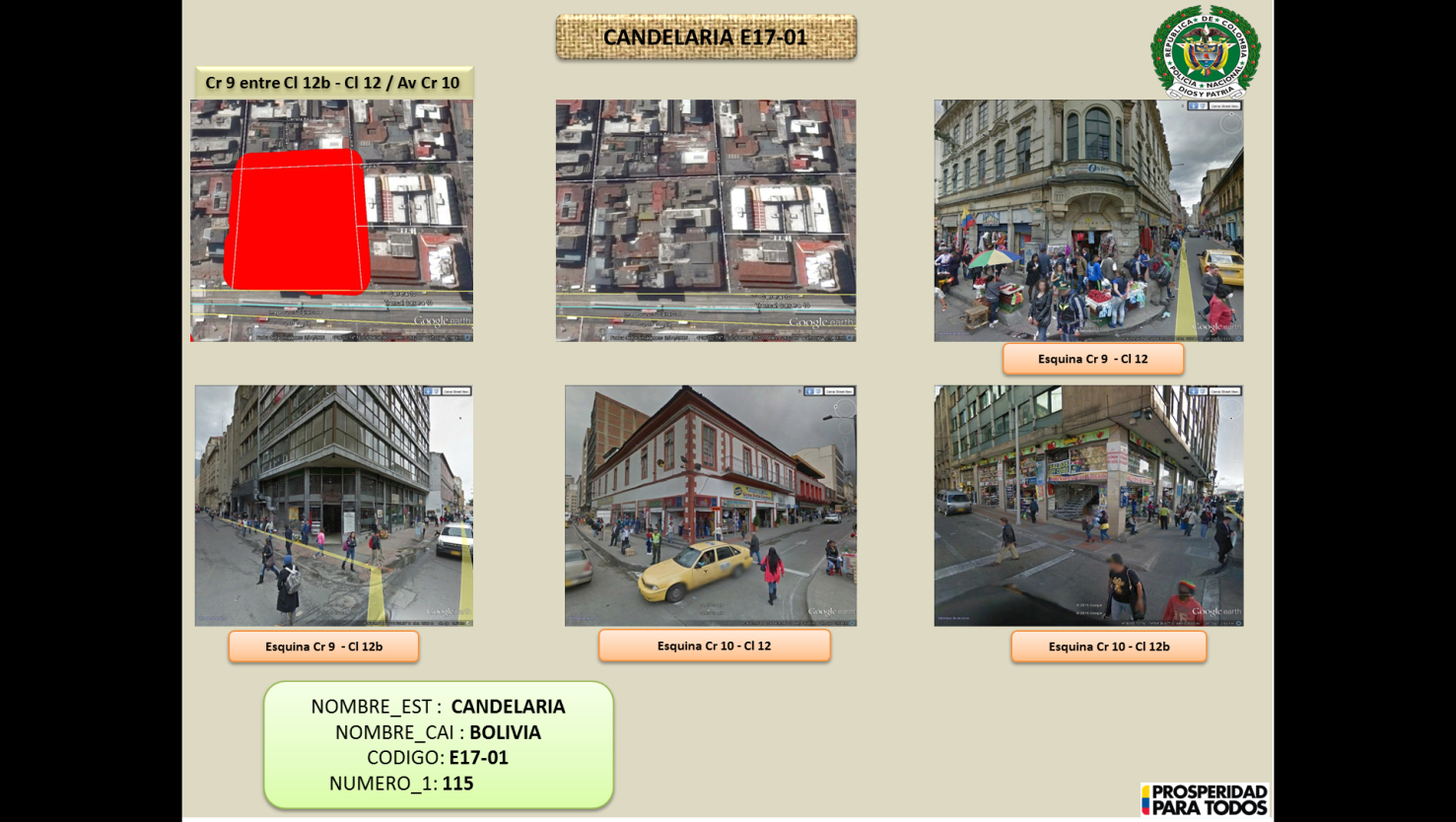

Supplement: S2 Fig — (DOCX) [file pone.0200593.s002.docx]
