# Supplementary material for: Deterring delinquents with information. Evidence from a randomized poster campaign in Bogotá
Source: PLoS One. 2018 Jul 19;13(7):e0200593. doi: 10.1371/journal.pone.0200593 (PMC6053166; doi:10.1371/journal.pone.0200593)
Supplement: S1 Table — (DOCX) [file pone.0200593.s003.docx]

**S1 Table. Balance tables**

**Balance between treatment and control areas during pre-treatment phase**

|  | control | treated | p-value |
| --- | --- | --- | --- |
| Total Crime | 3.49 | 4.38 | 0.20 |
| Total 123 Calls | 13.51 | 17.01 | 0.12 |
| Total Minor Wrongdoings | 3.87 | 2.77 | 0.33 |
| Premeditated Crime | 2.27 | 3.05 | 0.18 |
| Spontaneous Crime | 0.51 | 0.49 | 0.92 |
| 123 Calls for Attack | 10.74 | 13.16 | 0.20 |
| 123 Calls for Theft | 2.77 | 3.86 | 0.05 |
| Catchment Area Crime | 22.86 | 19.70 | 0.11 |
| Catchment Area 123 Calls | 122.23 | 104.13 | 0.25 |
| Catchment Area Minor Wrongdoings | 22.47 | 17.79 | 0.40 |
| Distance from City Center | 6975.79 | 6325.64 | 0.41 |
| Randomization Blocks | 39.00 | 39.00 | 1.00 |
| Pedestrians | 27.78 | 40.76 | 0.00 |
| N | 77 | 77 |  |

**Balance between survey respondents of treatment and control areas**

|  | control | treated | p-value |
| --- | --- | --- | --- |
| Sex | 1.50 | 1.49 | 0.81 |
| Age | 33.78 | 33.98 | 0.81 |
| Resident | 1.24 | 1.24 | 0.91 |
| Education | 3.02 | 3.16 | 0.03 |
| Socio-Economic Strata | 2.67 | 2.63 | 0.50 |
| Media Consumption | 1.50 | 1.70 | 0.00 |
| N | 308 | 308 |  |
