# Supplementary material for: Deterring delinquents with information. Evidence from a randomized poster campaign in Bogotá
Source: PLoS One. 2018 Jul 19;13(7):e0200593. doi: 10.1371/journal.pone.0200593 (PMC6053166; doi:10.1371/journal.pone.0200593)
Supplement: S2 Table — (DOCX) [file pone.0200593.s004.docx]

**S2 Table. T-test tables for hypotheses 1 to 5**

**Regression table for hypothesis 1**

|  | (1) | (2) | (3) |
| --- | --- | --- | --- |
|  | Total Crime | Total 123 Calls | Total Minor Wrongdoings |
| Treatment | -0.671 (0.508) | -0.933 (1.128) | 0.492 (0.894) |
| Distance from City Center | 0.0000538 (0.0000573) | -0.000153 (0.000127) | 0.000136 (0.000101) |
| Randomization Blocks | -0.00937 (0.0118) | 0.0155 (0.0262) | -0.0118 (0.0207) |
| Pedestrians | 0.0224 (0.0141) | -0.00582 (0.0313) | 0.00989 (0.0248) |
| Constant | -1.229 (0.922) | 1.924 (2.047) | -1.710 (1.622) |
| Observations | 154 | 154 | 154 |

Standard errors in parentheses

* p<0.10, ** p<0.05, *** p<0.010

**T-test table for hypothesis 1**

|  | Control | Treated | Difference | p-value |
| --- | --- | --- | --- | --- |
| Total Crime | -0.60 | -1.01 | 0.42 | 0.19 |
| Total 123 Calls | 1.30 | 0.39 | 0.91 | 0.20 |
| Total Minor Wrongdoings | -0.95 | -0.42 | -0.53 | 0.74 |

**Regression table for Hypothesis 2**

|  | (1) | (2) | (3) | (4) |
| --- | --- | --- | --- | --- |
|  | Premeditated Crime | Spontaneous Crime | 123 Calls for Theft | 123 Calls for Attack |
| Treatment | -0.710* (0.364) | 0.00921 (0.160) | -0.617 (0.570) | -0.316 (0.966) |
| Distance from City Center | 0.0000215 (0.0000411) | 0.0000269 (0.0000181) | -0.000121* (0.0000644) | -0.0000318 (0.000109) |
| Randomization Blocks | -0.00801 (0.00844) | 0.00175 (0.00372) | 0.00159 (0.0132) | 0.0139 (0.0224) |
| Pedestrians | 0.0127 (0.0101) | -0.000363 (0.00444) | -0.00453 (0.0158) | -0.00129 (0.0268) |
| Constant | -0.360 (0.661) | -0.376 (0.291) | 1.780* (1.036) | 0.144 (1.755) |
| Observations | 154 | 154 | 154 | 154 |

Standard errors in parentheses

* p<0.10, ** p<0.05, *** p<0.010

**T-test table for hypothesis 2**

|  | Control | Treated | Difference | p-value |
| --- | --- | --- | --- | --- |
| Premeditated Crime | -0.17 | -0.73 | 0.56 | 0.05 |
| Spontaneous Crime | -0.13 | -0.14 | 0.01 | 0.47 |
| Theft | 0.87 | 0.27 | 0.60 | 0.13 |
| Attack | 0.43 | 0.12 | 0.31 | 0.36 |

**Regression table for hypothesis 3**

|  | (1) | (2) | (3) |
| --- | --- | --- | --- |
|  | First Month Crime | Second Month Crime | Third Month Crime |
| Treatment | -0.365* (0.216) | -0.0338 (0.232) | -0.180 (0.224) |
| Distance from City Center | 0.0000280 (0.0000243) | 0.0000385 (0.0000262) | -0.00000332 (0.0000253) |
| Randomization Blocks | 0.00419 (0.00500) | -0.00225 (0.00539) | -0.00696 (0.00520) |
| Pedestrians | 0.0109* (0.00598) | 0.000867 (0.00644) | 0.0130** (0.00621) |
| Constant | -0.665* (0.391) | -0.443 (0.422) | -0.331 (0.407) |
| Observations | 154 | 154 | 154 |

Standard errors in parentheses

* p<0.10, ** p<0.05, *** p<0.010

**T-test table for hypothesis 3**

|  | Control | Treated | Difference | p-value |
| --- | --- | --- | --- | --- |
| First Month Crime | -0.00 | -0.25 | 0.24 | 0.12 |
| Second Month Crime | -0.24 | -0.29 | 0.05 | 0.41 |
| Third Month Crime | -0.26 | -0.27 | 0.01 | 0.48 |

**Regression table for hypothesis 4**

|  | (1) | (2) | (3) |
| --- | --- | --- | --- |
|  | Catchment Area Crime | Catchment Area 123 Calls | Catchment Area Minor Wrongdoings |
| Treatment | 0.412 (1.224) | 3.378 (3.360) | 1.250 (4.105) |
| Distance from City Center | 0.0000627 (0.000138) | 0.000363 (0.000379) | -0.000985** (0.000463) |
| Randomization Blocks | 0.0411 (0.0284) | 0.109 (0.0779) | 0.0000264 (0.0952) |
| Pedestrians | 0.0744** (0.0340) | -0.0180 (0.0932) | 0.0955 (0.114) |
| Constant | -5.068** (2.223) | -6.209 (6.100) | -0.721 (7.453) |
| Observations | 154 | 154 | 154 |

Standard errors in parentheses

* p<0.10, ** p<0.05, *** p<0.010

**T-test table for hypothesis 4**

|  | Control | Treated | Difference | p-value |
| --- | --- | --- | --- | --- |
| Catchment Area Crime | -0.96 | 0.38 | -1.34 | 0.87 |
| Catchment Area 123 Calls | 0.08 | 2.99 | -2.91 | 0.82 |
| Catchment Area Minor Wrongdoings | -4.94 | -1.81 | -3.13 | 0.79 |

**Regression table for hypothesis 5**

|  | (1) | (2) | (3) |
| --- | --- | --- | --- |
|  | Trust in Police | Security Feeling | Police Performance |
| Treatment | 0.0885 (0.104) | 0.102 (0.0629) | 0.0410 (0.0793) |
| Sex | -0.0866 (0.103) | 0.0117 (0.0622) | -0.268*** (0.0785) |
| Age | 0.0102* (0.00528) | -0.00998*** (0.00319) | 0.0138*** (0.00403) |
| Resident | -0.174 (0.121) | -0.527*** (0.0731) | 0.0473 (0.0919) |
| Education | 0.354*** (0.0722) | -0.0330 (0.0436) | 0.221*** (0.0550) |
| Socio-Economic Strata | -0.0197 (0.0833) | 0.324*** (0.0502) | 0.0692 (0.0634) |
| Media Consumption | -0.0954 (0.0772) | -0.106** (0.0467) | 0.153*** (0.0587) |
| Constant | 2.199*** (0.425) | 3.343*** (0.257) | 1.418*** (0.324) |
| Observations | 605 | 604 | 604 |

Standard errors in parentheses

* p<0.10, ** p<0.05, *** p<0.010

**T-test table for hypothesis 5**

|  | Control | Treated | Difference | p-value |
| --- | --- | --- | --- | --- |
| Trust in Police | 3.07 | 3.20 | -0.13 | 0.10 |
| Security Perception | 2.98 | 3.04 | -0.06 | 0.18 |
| Police Performance | 2.62 | 2.73 | -0.11 | 0.09 |
