# Supplementary material for: Deterring delinquents with information. Evidence from a randomized poster campaign in Bogotá
Source: PLoS One. 2018 Jul 19;13(7):e0200593. doi: 10.1371/journal.pone.0200593 (PMC6053166; doi:10.1371/journal.pone.0200593)
Supplement: S1 File — (DOCX) [file pone.0200593.s005.docx]

**S1 File. Full original survey in Spanish and English**

| **[ID. Número de cuestionario]** |  |  |
| --- | --- | --- |
| **[CUADRANTE. Número de cuadrante:]** |  |  |
| **[MANZANA. Número de manzana:]** |  |  |
| **[SALUDAR LIBREMENTE USANDO ESTE TEXTO]**  BUENOS DÍAS, SOY [**decir nombre**]. ESTOY HACIENDO UN ESTUDIO SOBRE PERCEPCIÓN DE SEGURIDAD EN BOGOTÁ PARA SUGERIRLE A LA POLICÍA NACIONAL COMO DISMINUIR LOS NIVELES DE CRIMINALIDAD. LE QUISIERA HACER UNAS POCAS PREGUNTAS LO QUE LE TOMARÍA 5 MINUTOS DE SU TIEMPO. SU OPINIÓN ES MUY IMPORTANTE PARA NOSOTROS Y PARA MEJORAR LA SEGURIDAD DE LOS BOGOTANOS. | |  |
| **FILTRO1.** ¿Está usted frecuentemente en este barrio? **[por lo menos una vez a la semana]**  **Si🡪Continúe con FILTRO2.**  **No🡪 Agradezca al entrevistado y termine la entrevista** | | |
| **FILTRO2**. **[No preguntar a personas claramente mayores de 18]** ¿Tiene más de 18 años?  **Sí🡪Continúe leyendo consentimiento**  **No🡪Agradezca al entrevistado y termine la entrevista** | |  |
| **[LEER ESTE CONSENTIMIENTO INFORMADO ANTES DE INICIAR CON LAS PREGUNTAS]**  ANTES DE EMPEZAR CON EL RESTO DE PREGUNTAS, QUIERO ASEGURARLE QUE SU PARTICIPACIÓN EN EL ESTUDIO ES VOLUNTARIA. LAS RESPUESTAS QUE USTED PROPORCIONE SERÁN COMPLETAMENTE CONFIDENCIALES Y NO REGISTRAMOS SU NOMBRE O IDENTIDAD. ¿DESEA PARTICIPAR? | |  |

| **Q1.** Género **[ANOTAR, NO PREGUNTE]**: (1) Hombre (2) Mujer | | | | | | | | | | | | | | | | | |  |
| --- | --- | --- | --- | --- | --- | --- | --- | --- | --- | --- | --- | --- | --- | --- | --- | --- | --- | --- |
| **EDAD.** ¿Qué edad tiene? **[en años]** (8888) NS (9888) NR | | | | | | | | | | | | | | | | | |  |
| **RESIDENTE.** ¿Reside en este barrio en el que nos encontramos ahora?  (1) Sí (2) No (88) NS (98) NR | | | | | | | | | | | | | | | | | |  |
| **TRABAJADOR.** ¿Trabaja en este barrio aquí? (1) Sí (2) No (88) NS (98) NR | | | | | | | | | | | | | | | | | |  |
| **EDU.** ¿Cuál es su nivel educativo más alto alcanzado? (1) ninguno (2) primaria  (3) secundaria (4) técnico/tecnológico (5) profesional (6) posgrado | | | | | | | | | | | | | | | | | |  |
| **ESTRATO.** ¿Cuál es su estrato? (1) (2) (3) (4) (5) (6) | | | | | | | | | | | | | | | | | |  |
| **GI0.** ¿Con qué frecuencia sigue las noticias, ya sea en televisión, radio, periódicos o Internet? **[Leer]** (1) Diariamente (2) Algunas veces a la semana (3) Algunas veces al mes (4) Rara vez (5) Nunca **[No leer]** (88) NS **[No leer]** (98) NR | | | | | | | | | | | | | | | | | |  |
| Cambiando de tema, le voy a hacer una serie de preguntas, y le voy a pedir que para darme su respuesta utilice los números entre 1 y 7, donde 1 significa NADA y 7 significa MUCHO. Por ejemplo, si yo le preguntara hasta qué punto le gusta ver televisión, si a usted no le gusta ver nada, elegiría un 1 o 2. Si por el contrario le gusta mucho ver televisión me diría el número 7 o 6. Si su opinión está entre nada y mucho elegiría un número entre 1 y 7, por ejemplo 3, 4 o 5. Entonces, ¿hasta qué punto le gusta a usted ver televisión? Dígame el número. ***[Asegúrese que el entrevistado entienda correctamente]***. | | | | | | | | | | | | | | | | | | |
| 1 | | | 2 | 3 | | 4 | | | | 5 | | 6 | | | 7 | 88 | | 98 |
| **Nada** | | | | | | | | **Mucho** | | | | | | | | **No sabe** | | **No responde** |
| Ahora le voy a pedir que para darme su respuesta utilice los números entre 1 y 7. Recuerde que puede utilizar cualquier número intermedio. | | | | | | | | | | | | | | | | | | |
| **Anotar el número 1-7, 88 para los que NS y 98 para los NR** | | | | | | | | | | | | | | | | | | |
| **B10A.** ¿Hasta qué punto tiene confianza en el sistema de justicia? | | | | | | | | | | | | | | | | | |  |
| **B14**. ¿Hasta qué punto tiene confianza usted en el Gobierno Nacional? | | | | | | | | | | | | | | | | | |  |
| **B18**. ¿Hasta qué punto tiene confianza usted en la Policía? | | | | | | | | | | | | | | | | | |  |
| **B32**. ¿Hasta qué punto tiene usted confianza en la alcaldía de Bogotá? | | | | | | | | | | | | | | | | | |  |
| **VIC1EXT.** Ahora, cambiando el tema, ¿ha sido usted víctima de algún acto de delincuencia en los últimos 3 meses? Es decir, ¿ha sido usted víctima de un robo, hurto, agresión, fraude, extorsión, amenazas o algún otro tipo de acto delincuencial en los últimos 3 meses? (1) Sí [Siga] (2) No **[Pasar a AOJ11]**  (88) NS **[Pasar a AOJ11]** (98) NR **[Pasar a AOJ11]** | | | | | | | | | | | | | | | | | |  |
| **AOJ1.** ¿Denunció el hecho del que fue víctima a alguna institución?  (1) Sí (2) No (88) NS (98) NR | | | | | | | | | | | | | | | | | |  |
| **AOJ11.** Hablando de este barrio de por aquí y pensando en la posibilidad de ser víctima de un asalto o robo, ¿usted se siente muy seguro(a), algo seguro(a), algo inseguro(a) o muy inseguro(a)?  (1) Muy seguro(a) (2) Algo seguro(a) (3) Algo inseguro(a) (4) Muy inseguro(a)  (88) NS (98) NR | | | | | | | | | | | | | | | | | |  |
| **AOJ12A.** Si usted fuera víctima de un robo o asalto, ¿cuánto confiaría que la policía capturaría a la persona responsable? **[Leer alternativas]** Confiaría…  (1) Mucho (2) Algo (3) Poco (4) Nada (88) NS (98) NR | | | | | | | | | | | | | | | | | |  |
| **AOJ12.** Y si fuera víctima de un robo o asalto, ¿cuánto confiaría que el sistema judicial castigue al culpable? **[Leer alternativas]** Confiaría…  (1) Mucho (2) Algo (3) Poco (4) Nada (88) NS (98) NR | | | | | | | | | | | | | | | | | |  |
| **PESE2.** ¿Considera usted que el nivel de violencia actual en este barrio de por aquí es mayor, igual, o menor que el de hace 12 meses?  (1) Mayor (2) Igual (3) Menor (88) NS (98) NR | | | | | | | | | | | | | | | | | |  |
| **DESEMP.** ¿Cómo califica la labor que desempeñan en materia de seguridad, las siguientes entidades? Siendo 1 pésimo, 2 mala, 3 regular, 4 buena, y 5 excelente **[Lea cada opción y califique sólo aquellos que conoce]** | | | | | | | | | | | | | | | | |  |  |
| Alcaldía de Bogotá | | | 1 | | 2 | | 3 | | 4 | | 5 | (88) NS (98) NR | | | |  |  |  |
| Gobierno Nacional | | | 1 | | 2 | | 3 | | 4 | | 5 | (88) NS (98) NR | | | |  |  |  |
| Policía Nacional | | | 1 | | 2 | | 3 | | 4 | | 5 | (88) NS (98) NR | | | |  |  |  |
| **IT1.** Ahora, hablando de la gente de por aquí, ¿diría que la gente de por aquí es muy confiable, algo confiable, poco confiable o nada confiable?  (1) Muy confiable (2) Algo confiable (3) Poco confiable (4) Nada confiable (88) NS (98) NR | | | | | | | | | | | | | | | | | |  |
| **EXPERIMENT1.** ¿En los últimos meses usted ha visto afiches en esta manzana aquí indicando el número de capturados por la Policía Nacional? **[NO SEÑALE AFICHE]**  (1) Sí **[Siga con Experiment2]**  (2) No **[Termine entrevista]**  (88) NS **[Termine entrevista]** (98) NR **[Termine entrevista]** | | | | | | | | | | | | | | | |  | |  |
| **EXPERIMENT2.** ¿Le gustaron o le molestaron estos afiches de la Policía?  (1) Me gustaron (2) Me molestaron 3) **[NO LEER]** No me gustó ni me molestó (88) NS (98) NR | | | | | | | | | | | | | | | |  | |  |

ESTAS SON TODAS LAS PREGUNTAS. ¡MUCHAS GRACIAS POR SU VALIOSA COLABORACIÓN!

**[Siguientes preguntas para encuestador]**

| **[FECHA. Fecha de entrevista Día: Mes: Año: 2016]** |
| --- |

| **[Hora en la cual terminó la entrevista]** | | **_________ : _________** |
| --- | --- | --- |
| **[TI. Duración de la entrevista en minutos]** | |  |
| **[INTID. Nombre del entrevistador/a]** |  | |

| *Yo juro que esta entrevista fue llevada a cabo con la persona indicada.* |
| --- |
| *Firma del entrevistador_________________________________ Fecha ____ /_____ /_____*  *Firma del supervisor de campo _______________________________________* |

| **[ID. Number of questionnaire]** |  |  |
| --- | --- | --- |
| **[CUADRANTE. Number of “cuadrante”:]** |  |  |
| **[MANZANA. Number of “manzana”:]** |  |  |
| **[Greet people freely, in accordance with following text]**  HELLO, I AM [**SAY YOUR NAME**]. I AM DOING A STUDY ABOUT SECURITY PERCEPTION IN BOGOTÁ WITH THE OBJECTIVE TO HELP THE NATIONAL POLICE TO DIMINISH LEVELS OF CRIME. I WOULD LIKE TO ASK YOU A FEW QUESTIONS WHICH WOULD TAKE ABOUT FIVE MINUTES OF YOUR TIME. YOUR OPINION IS VERY IMPORTANT FOR US AND TO HELP IMPROVE THE SECURITY SITUATION OF PEOPLE LIVING IN BOGOTÁ. | |  |
| **FILTER1.** Are you frequently in this area? **[at least once a week]**  **Yes🡪Continue with FILTER2.**  **No🡪Thank the interviewee and finalize the interview** | | |
| **FILTER2**. **[Do not ask this to people who are clearly above 18]** Are you above 18  years of age?  **Yes🡪Continue reading informed consent**  **No🡪Thank the interviewee and finalize the interview** | |  |
| **[READ THIS INFORMED CONSENT PASSAGE BEFORE FURTHER QUESTIONS]**  BEFORE WE BEGIN WITH THE REST OF THE QUESTIONS, I WOULD LIKE TO ASSURE YOU THAT YOUR PARTICIPATION IN THIS STUDY IS VOLUNTARY. THE ANSWERS THAT YOU GIVE ARE GOING TO BE TREATED WITH FULL CONFIDENTIALITY AND NO REGISTER OF YOUR NAME OR IDENTITY WILL BE MADE. DO YOU WANT TO PARTICIPATE? | |  |

| **Q1.** Sex **[NOTE, DO NOT ASK]**: (1) Man (2) Woman | | | | | | | | | | | | | | | | | |  |
| --- | --- | --- | --- | --- | --- | --- | --- | --- | --- | --- | --- | --- | --- | --- | --- | --- | --- | --- |
| **AGE.** How old are you? **[in years]** (8888) NS (9888) NR | | | | | | | | | | | | | | | | | |  |
| **RESIDENT.** Do you reside in this area where we are now?  (1) Yes (2) No (88) NS (98) NR | | | | | | | | | | | | | | | | | |  |
| **WORKER.** Do you work in this area here? (1) Yes (2) No (88) NS (98) NR | | | | | | | | | | | | | | | | | |  |
| **EDU.** What is your highest level of education? (1) none (2) primary school (3) secondary school (4) technical education (5) university education (6) postgraduate education | | | | | | | | | | | | | | | | | |  |
| **SES.** What is your socio-economic strata? (1) (2) (3) (4) (5) (6) | | | | | | | | | | | | | | | | | |  |
| **GI0.** With what frequency do you follow the news, be it through TV, radio, newspaper or internet? **[Read]** (1) Daily (2) Several times a week (3) Several times a month (4) Rarely (5) Never **[Do not read]** (88) NS (98) NR | | | | | | | | | | | | | | | | | |  |
| Changing the topic, I am going to ask a series of questions and I will ask you to give your response indicating a number between 1 and 7, where 1 means not at all and 7 means a lot. For example, if I was to ask you how much you like to watch TV, and you didn’t like it all, you would say 1 or perhaps 2. If on the contrary, you like to watch TV a lot, you would say 7 or 6. If your opinion is somewhere in between not at all and a lot, you would choose a number between 1 and 7, for example 3, 4 or 5. So, ¿to what extent to you like to watch TV? Tell me the number, please. ***[Be sure that the interviewee understood correctly]***. | | | | | | | | | | | | | | | | | | |
| 1 | | | 2 | 3 | | 4 | | | | 5 | | 6 | | | 7 | 88 | | 98 |
| **Not at all** | | | | | | | | **A lot** | | | | | | | | **Don’t know** | | **No response** |
| Now I would like to ask you to give me your response only using the numbers between 1 and 7. Remember that you can use any number in between. | | | | | | | | | | | | | | | | | | |
| **Note numbers between 1-7, 88 NS and 98 NR** | | | | | | | | | | | | | | | | | | |
| **B10A.** To what extent do you have trust in the justice system? | | | | | | | | | | | | | | | | | |  |
| **B14**. To what extent do you have trust in the National Government? | | | | | | | | | | | | | | | | | |  |
| **B18**. To what extent to you have trust in the Police? | | | | | | | | | | | | | | | | | |  |
| **B32**. To what extent do you have trust in the Mayor’s office of Bogotá? | | | | | | | | | | | | | | | | | |  |
| **VIC1EXT.** Now, changing the topic, have you been a victim of some act of delinquency in the past three months? That means, have you been a victim of robbery, theft, aggression, fraud, extorsion, threats or any other type of delinquent act in the past three months? (1) Yes [follow] (2) No **[Go to AOJ11]**  (88) NS **[Go to AOJ11]** (98) NR **[Go to AOJ11]** | | | | | | | | | | | | | | | | | |  |
| **AOJ1.** Did you denounce this crime you were a victim of to any institution?  (1) Yes (2) No (88) NS (98) NR | | | | | | | | | | | | | | | | | |  |
| **AOJ11.** Speaking about this area around here and thinking of the possibility of being a victim of an assault or robbery, do you feel very safe, somewhat safe, somewhat unsafe, very unsafe?  (1) very safe (2) somewhat safe (3) somewhat unsafe (4) very unsafe  (88) NS (98) NR | | | | | | | | | | | | | | | | | |  |
| **AOJ12A.** If you were a victim of a robbery or assault, to what extent would you trust the police to arrest the responsible person? **[Read alternatives]** You would trust…  (1) A lot (2) Somewhat (3) Little (4) Not at all (88) NS (98) NR | | | | | | | | | | | | | | | | | |  |
| **AOJ12.** If you were a victim of a robbery or assault, to what extent would you trust the justice system to punish the responsible person? **[Read alternatives]** You would trust…  (1) A lot (2) Somewhat (3) Little (4) Not at all (88) NS (98) NR | | | | | | | | | | | | | | | | | |  |
| **PESE2.** Do you consider that the level of violence in this area around here is currently higher, the same or lower than 12 months ago?  (1) Higher (2) the same (3) lower (88) NS (98) NR | | | | | | | | | | | | | | | | | |  |
| **DESEMP.** How do you qualify the work of the following entities with regard to security? 1 very bad, 2 bad, 3 intermediate, 4 good, y 5 excellent **[Read all options and qualify only those they know]** | | | | | | | | | | | | | | | | |  |  |
| Bogotá mayor’s office | | | 1 | | 2 | | 3 | | 4 | | 5 | (88) NS (98) NR | | | |  |  |  |
| National government | | | 1 | | 2 | | 3 | | 4 | | 5 | (88) NS (98) NR | | | |  |  |  |
| National police | | | 1 | | 2 | | 3 | | 4 | | 5 | (88) NS (98) NR | | | |  |  |  |
| **IT1.** Now, speaking about people from around here, would you say that people are very trustworthy, somewhat trustworthy, little trustworthy, not trustworthy at all?  (1) Very trustworthy (2) Somewhat trustworthy (3) Little trustworthy (4) Not trustworthy at all (88) NS (98) NR | | | | | | | | | | | | | | | | | |  |
| **EXPERIMENT1.** Have you seen posters in this “manzana” here in the last few months indicating numbers of people arrested by the National Police? **[DO NOT SHOW POSTERS]**  (1) Yes **[Follow with Experiment2]**  (2) No **[Finalize interview]**  (88) NS **[Finalize interview]** (98) NR **[Finalize interview]** | | | | | | | | | | | | | | | |  | |  |
| **EXPERIMENT2.** Did you like or dislike the posters of the Police?  (1) Like (2) Dislike 3) **[DO NOT READ]** Neither/nor (88) NS (98) NR | | | | | | | | | | | | | | | |  | |  |

THESE ARE ALL THE QUESTIONS. THANK YOU VERY MUCH FOR YOUR COLLABORATION!

**[Following questions for the enumerator]**

| **[FECHA. Date of interview Day: Month: Year: 2016]** |
| --- |

| **[Hour in which interview finalized]** | | **_________ : _________** |
| --- | --- | --- |
| **[TI. Duration of interview]** | |  |
| **[INTID. Name of enumerator]** |  | |

| *I confirm that this interview was conducted with the indicated person.* |
| --- |
| *Enumerator signature______________________________Date____ /_____ /_____*  *Signature of field supervisor _______________________________________* |
