# Supplementary material for: Deterring delinquents with information. Evidence from a randomized poster campaign in Bogotá
Source: PLoS One. 2018 Jul 19;13(7):e0200593. doi: 10.1371/journal.pone.0200593 (PMC6053166; doi:10.1371/journal.pone.0200593)
Supplement: S2 File — (DOCX) [file pone.0200593.s006.docx]

**S2 File. Question framing for hypothesis 5**

Trust in police: „To what extent do you trust the police?” Answer options range from 1, none, to 7, a lot.

Security perception: „Talking about the neighborhood around here and thinking of becoming a victim to an assault or robbery, do you feel very secure, somewhat secure, somewhat insecure or very insecure?” Answer options from 1 to 4.

Police performance: „How do you assess the work of the Nacional Police in terms of security?” Answer options from 1, very bad, to 5, excellent.

NOTE: For the coefplot, all outcome variable were re-scaled to 0 to 1 scales.
