# Supplementary material for: Deterring delinquents with information. Evidence from a randomized poster campaign in Bogotá
Source: PLoS One. 2018 Jul 19;13(7):e0200593. doi: 10.1371/journal.pone.0200593 (PMC6053166; doi:10.1371/journal.pone.0200593)
Supplement: S3 File — (DOCX) [file pone.0200593.s007.docx]

**S3 File. List of crimes and minor wrongdoings**

***List of crimes included in total registered crimes (note that translations are literal and articles refer to Colombian penal code):***

ARTICULO 239. HURTO PERSONAS

Personal theft

ARTICULO 376. TRAFICO, FABRICACION O PORTE DE ESTUPEFACIENTES

Traffic, fabrication and carrying of illegal drugs

ARTICULO 111. LESIONES PERSONALES

Personal injury

ARTICULO 239. HURTO ENTIDADES COMERCIALES

Theft in commercial entity

ARTICULO 239. HURTO MOTOCICLETAS

Theft of motorbikes

ARTICULO 429. VIOLENCIA CONTRA SERVIDOR PUBLICO

Violence against public servant

ARTICULO 265. DANO EN BIEN AJENO

Damage to others’ goods

ARTICULO 229. VIOLENCIA INTRAFAMILIAR

Domestic violence

ARTICULO 365. FABRICACION, TRAFICO, PORTE O TENENCIA DE ARMAS DE

FUEGO, ACCESORIOS, PARTES O MUNICIONES

Traffic, fabrication and carrying of firearms, accessories, parts or ammunition

ARTICULO 109. HOMICIDIO CULPOSO (EN ACCIDENTE DE TRANSITO)

Manslaughter

ARTICULO 239. HURTO RESIDENCIAS

Theft of residential units

ARTICULO 239. HURTO AUTOMOTORES

Theft of cars

ARTICULO 120. LESIONES CULPOSAS (EN ACCIDENTE DE TRANSITO)

Careless injury

ARTICULO 103. HOMICIDIO

Homicide

ARTICULO 347. AMENAZAS

Threat

ARTICULO 208. ACCESO CARNAL ABUSIVO CON MENOR DE 14 ANOS

Rape of minor under the age of 14

***List of minor wrongdoings (contravenciones):***

Art.80 No. 1,2 y 4. OCUPACION ESPACIO PUBLICO POR VEHICULOS, VENTAS AMBULANTES

Occupation of public space

Art. 25. No. 10, NO UTILIZAR BANOS Y NO HACERLO EN SITIOS PUBLICOS

Not using bathrooms

Art.12,14, Art. 15,12. RINAS O ESCANDALO. AGREDIR FISICA O VERBALMENTE A VECINO ALGUNO

A QUIEN SEA AMONESTADO EN PRIVADO O REPRENDIDO EN AUDIENCIA PUBLICA

AL QUE EN VIA PUBLICA RINA O AMENACE A OTROS

Minor aggression

Art. 27 Numeral 3.2 Y 3.5 VENDER O CONSUMIR BEBIDAS EMBRIAGANTES ZONA COMUN ESPACIO PUBLICO.

Alcohol use in public

Art. 12. No. 16. ACTIVIDAD CONTAMINE AMBIENTE OCASIONE OLORES Y RUIDOS PERTURBEN LA TRANQUILIDAD

PERTURBAR TRANQUILIDAD EN RECINTO DE OFICINA PUBLICA,O DURANTE ESPECTUCULOS O REUNIONES PUBLICAS

Noise or smell contamination.

Art.83 No. 1, 2 y 3. OCUPACION ESPACIO PUBLICO CON RESIDUOS EL ARROJAR Y SU RECOLECCION

A QUIEN HAYA SIDO AMONESTADO EN PRIVADO O REPRENDIDO EN AUDIENCIA PUB

Occupation of public space with waste

ART. 111. No.7, INVASION ESPACIO, ESTABLECIMIENTO PUBLICO

Invasion of public space

Art.100 No. 15, 16,17y 18. CICLO RUTA NO UTILIZAR PARA: MOTOS U OTRO TIPO DE VEHICULO PARA PASEAR PERROS, VENTA ESTACIONARIA EN LOS PUENTES LLEVAR LA BICICLETA EN LA MANO.

CUANDO SE QUEBRANTE HORARIO DE SERVICIO SENALADO POR LA POLICIA LOCAL

Not using cycle path in correct way

Art. 82 No. 2, 3, 5. CONTAMINACION AUDITIVA Y SONORA

Noise contamination
